# Supplementary material for: Octa-coordinated alkaline earth metal–dinitrogen complexes M(N2)8 (M=Ca, Sr, Ba)
Source: Nat Commun. 2019 Jul 29;10:3375. doi: 10.1038/s41467-019-11323-5 (PMC6662891; doi:10.1038/s41467-019-11323-5)
Supplement: Supplementary file 1 — Supplementary Information [file 41467_2019_11323_MOESM1_ESM.doc]

Octa-Coordinated Alkaline Earth-Dinitrogen Complexes M(N_2_)_8_ (M = Ca, Sr, Ba)

Wang et al.

**Supplementary Figures**

**Supplementary Figure 1**. Infrared absorption spectra of strontium dinitrogen complexes in the 2300-1950 cm^-1^ region from co-deposition of laser-evaporated strontium atoms with 0.5% N_2_ in neon. a) 30 min of sample deposition at 4 K, b) after annealing at 10 K, c) after annealing at 12 K, d) after 15 min of UV-visible light irradiation.

**Supplementary Figure 2**. Infrared absorption spectra of calcium dinitrogen complexes in the 2300-1950 cm^-1^ region from co-deposition of laser-evaporated calcium atoms with 0.1% N_2_ in neon. a) 30 min of sample deposition at 4 K, b) after annealing at 10 K, c) after annealing at 13 K, d) after 15 min of visible light irradiation, and e) after 15 min of UV-visible light irradiation.

**Supplementary Figure 3**. Infrared absorption spectra of barium dinitrogen complexes in the 2200-1980 cm^-1^ region from co-deposition of laser-evaporated barium atoms with isotopic-substituted N_2_ in neon. a) 0.5% ^14^N_2_, b) 0.25% ^14^N_2_ + 0.25% ^15^N_2_, c) 0.5%^15^N_2_.

**Supplementary Figure 4**. Mass spectrum of the calcium dinitrogen cation complexes in the m/z range of 20-350 from pulsed laser evaporation of a calcium metal target in expansion of helium seeded with dinitrogen.

**Supplementary Figure 5**. Mass spectrum of the strontium dinitrogen cation complexes in the m/z range of 50-350 from pulsed laser evaporation of a strontium metal target in expansion of helium seeded with dinitrogen.

**Supplementary Figure 6**. Mass spectrum of the barium dinitrogen cation complexes in the m/z range of 100-500 from pulsed laser evaporation of a barium metal target in expansion of helium seeded with dinitrogen.

**Supplementary Figure 7**. Infrared photodissociation spectra of the (a) Ca(N_2_)_8_^+^ and (b) Sr(N_2_)_8_^+^ complexes in the 2000-2400 cm^-1^ region.

| 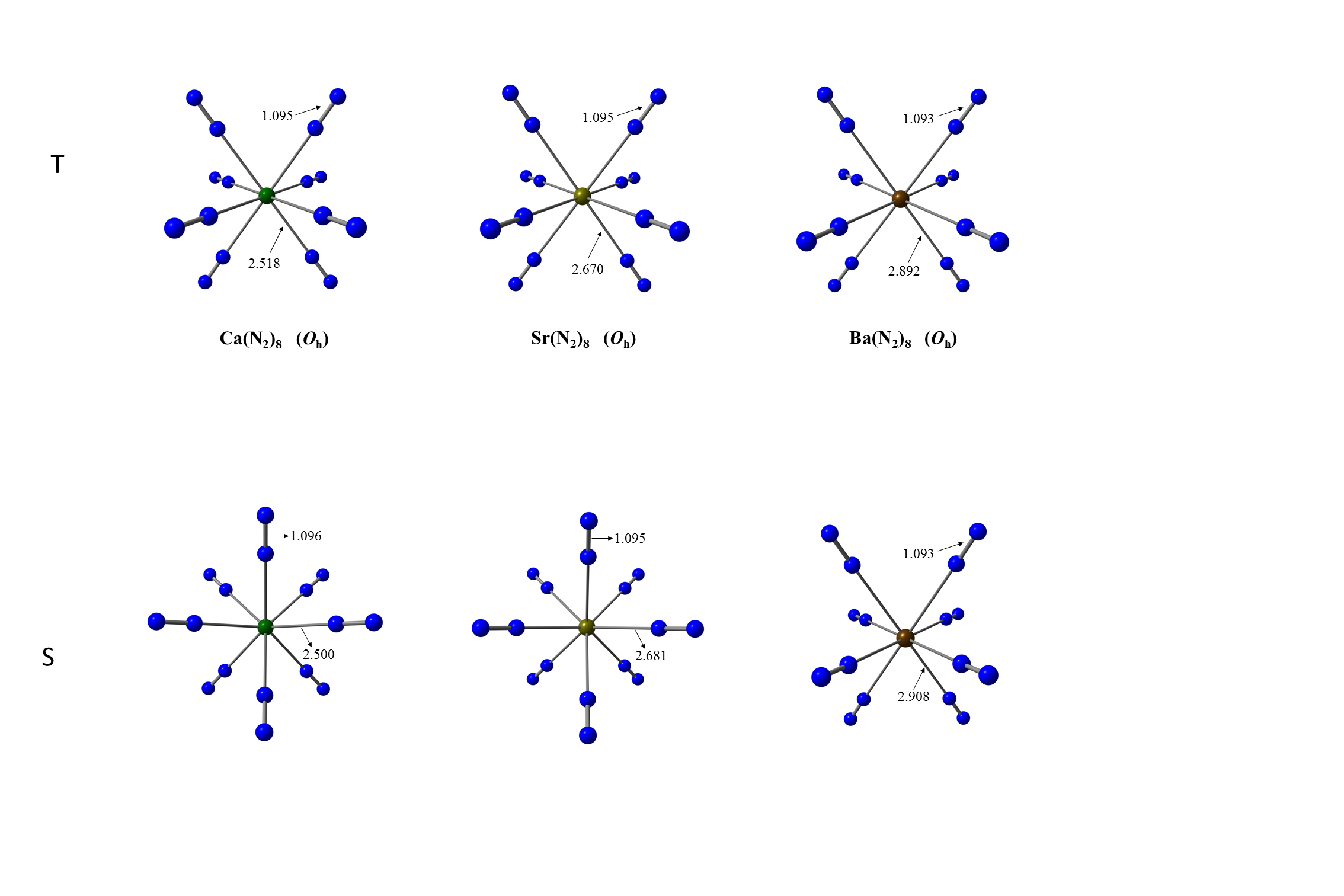  0.0  Ca(N_2_)_8_ (*O*_h_, T) | 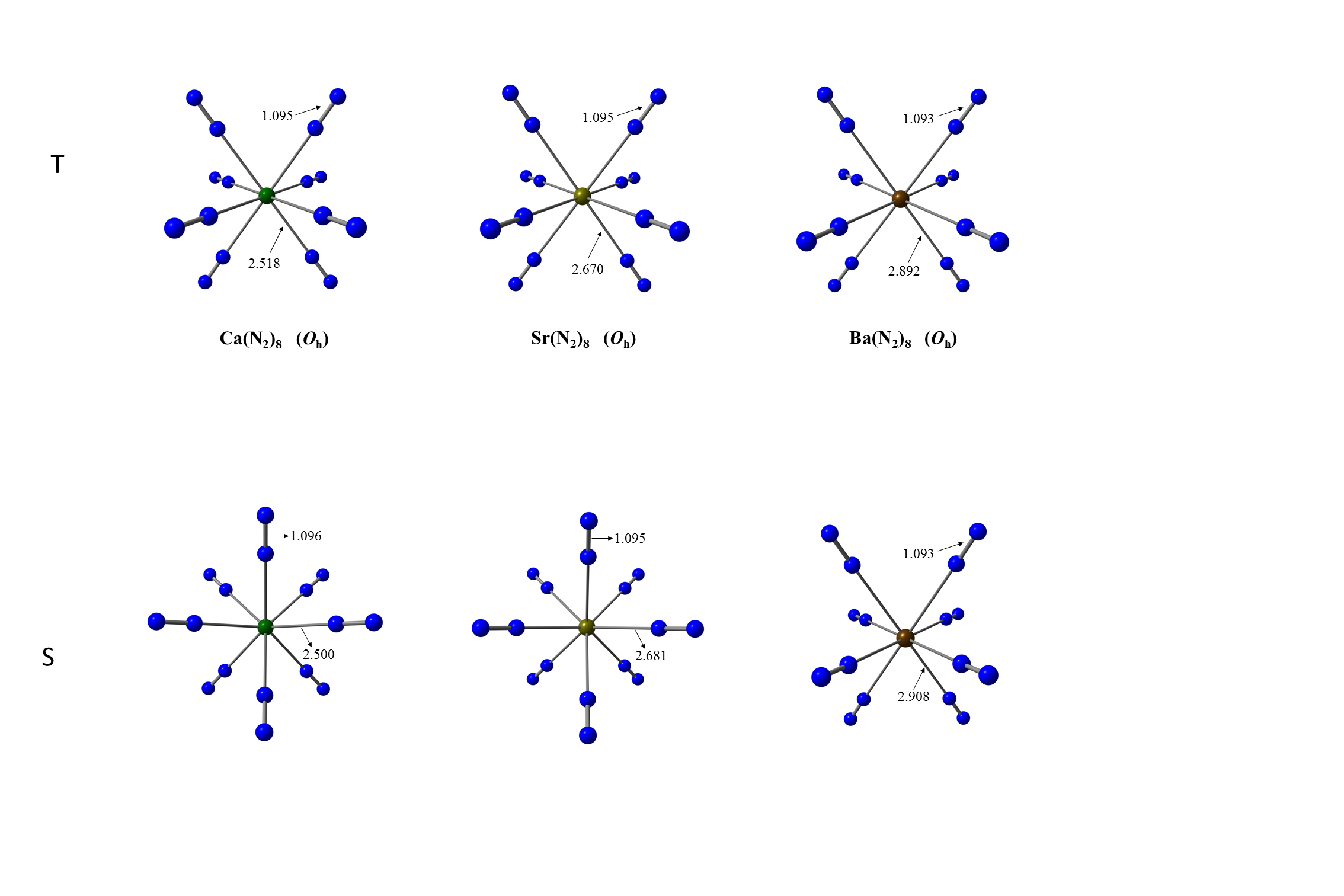  8.4  Ca(N_2_)_8_ (*D*_4_*_d_*, S) |
| --- | --- |
| 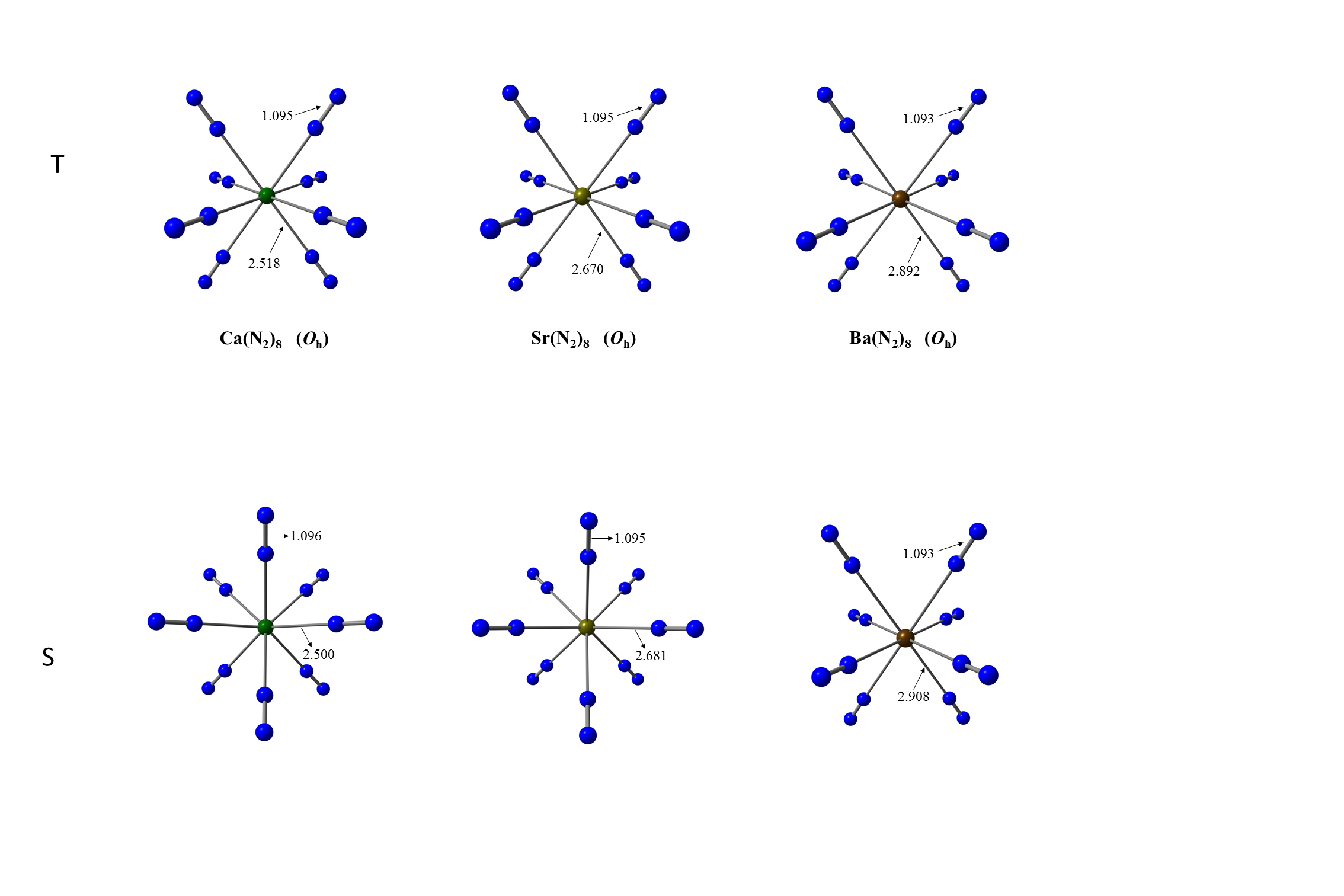  0.0  Sr(N_2_)_8_ (*O*_h_, T) | 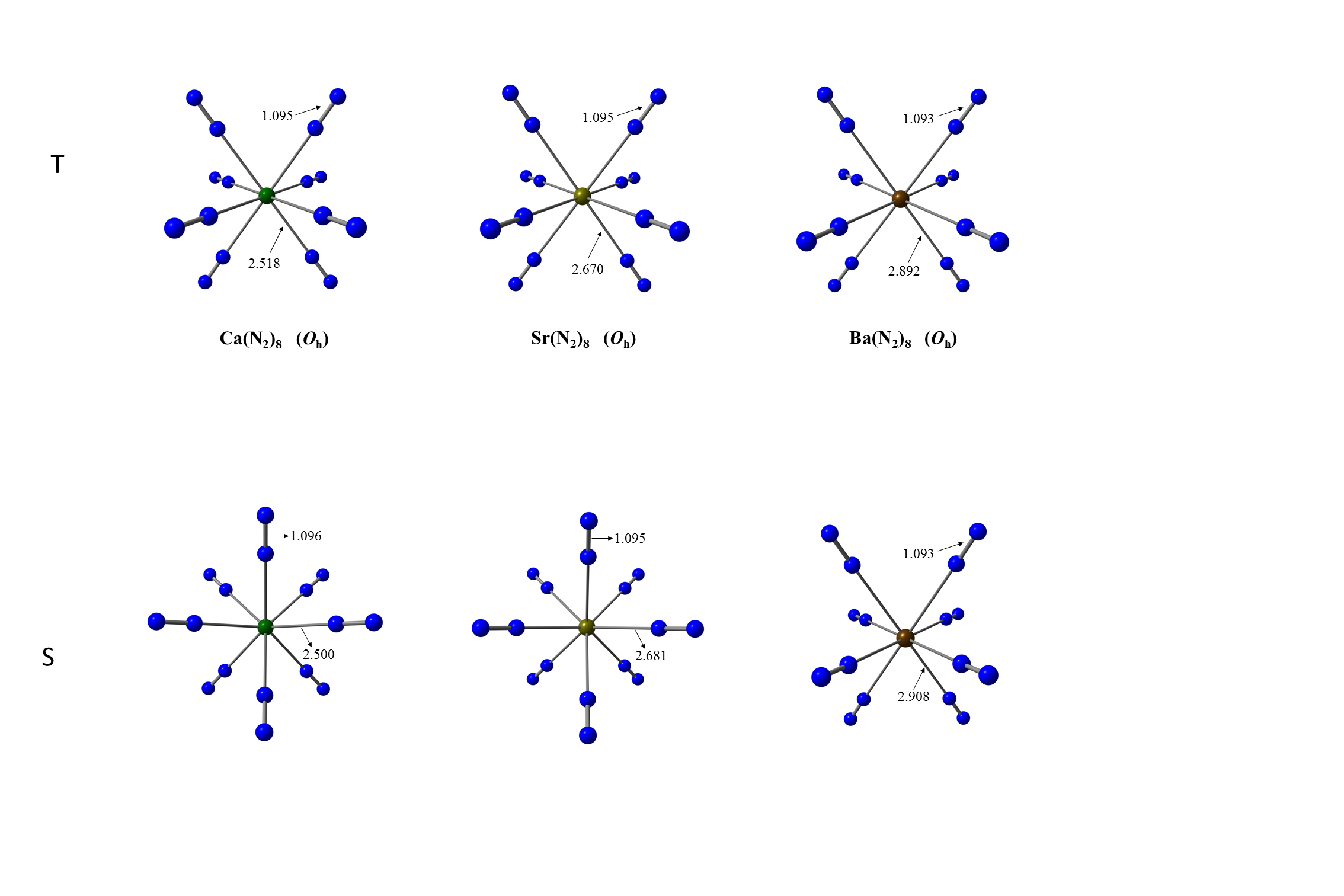  8.4  Sr(N_2_)_8_ (*D*_4_*_d_*, S) |
| 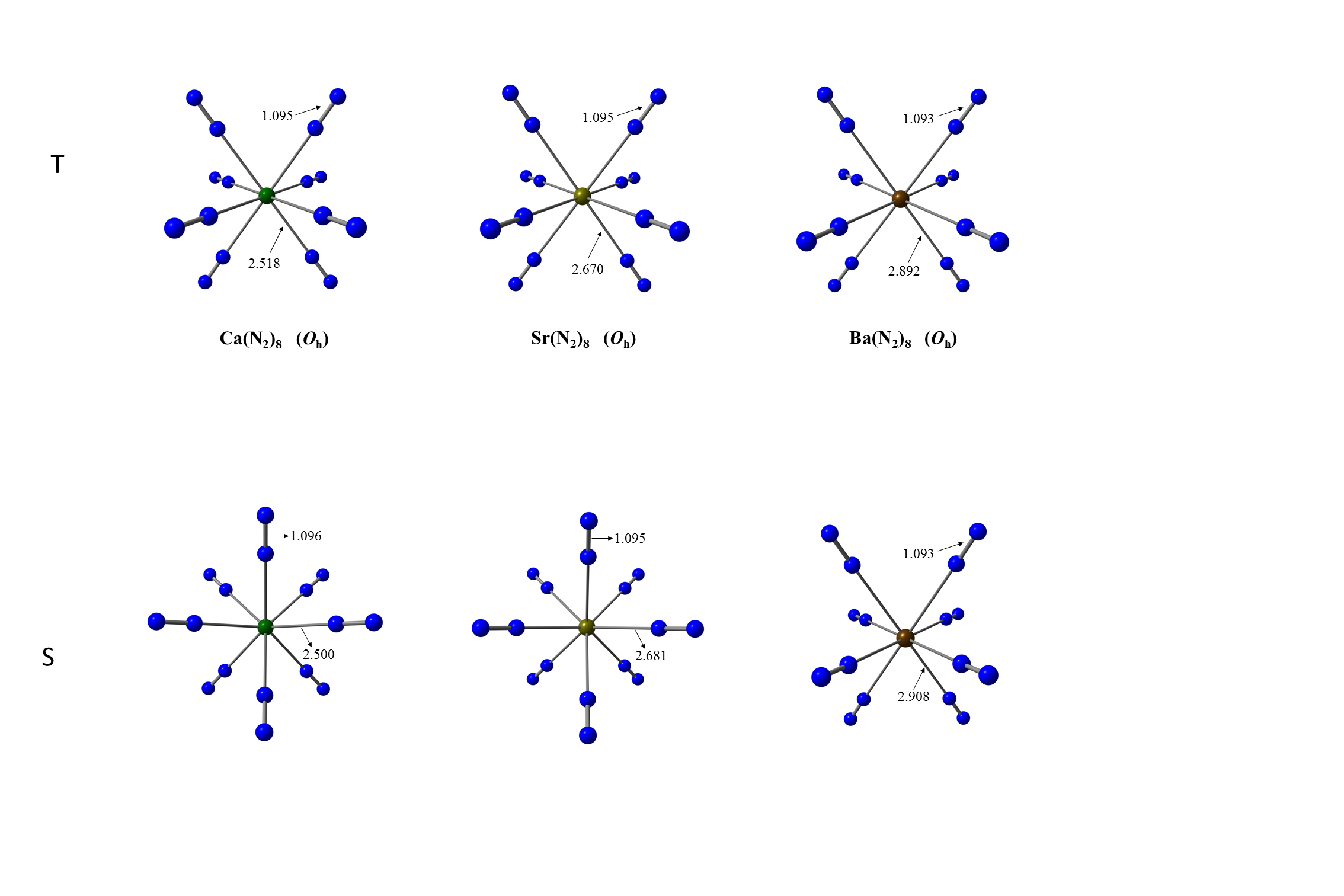  0.0  Ba(N_2_)_8_ (*O*_h_, T) | 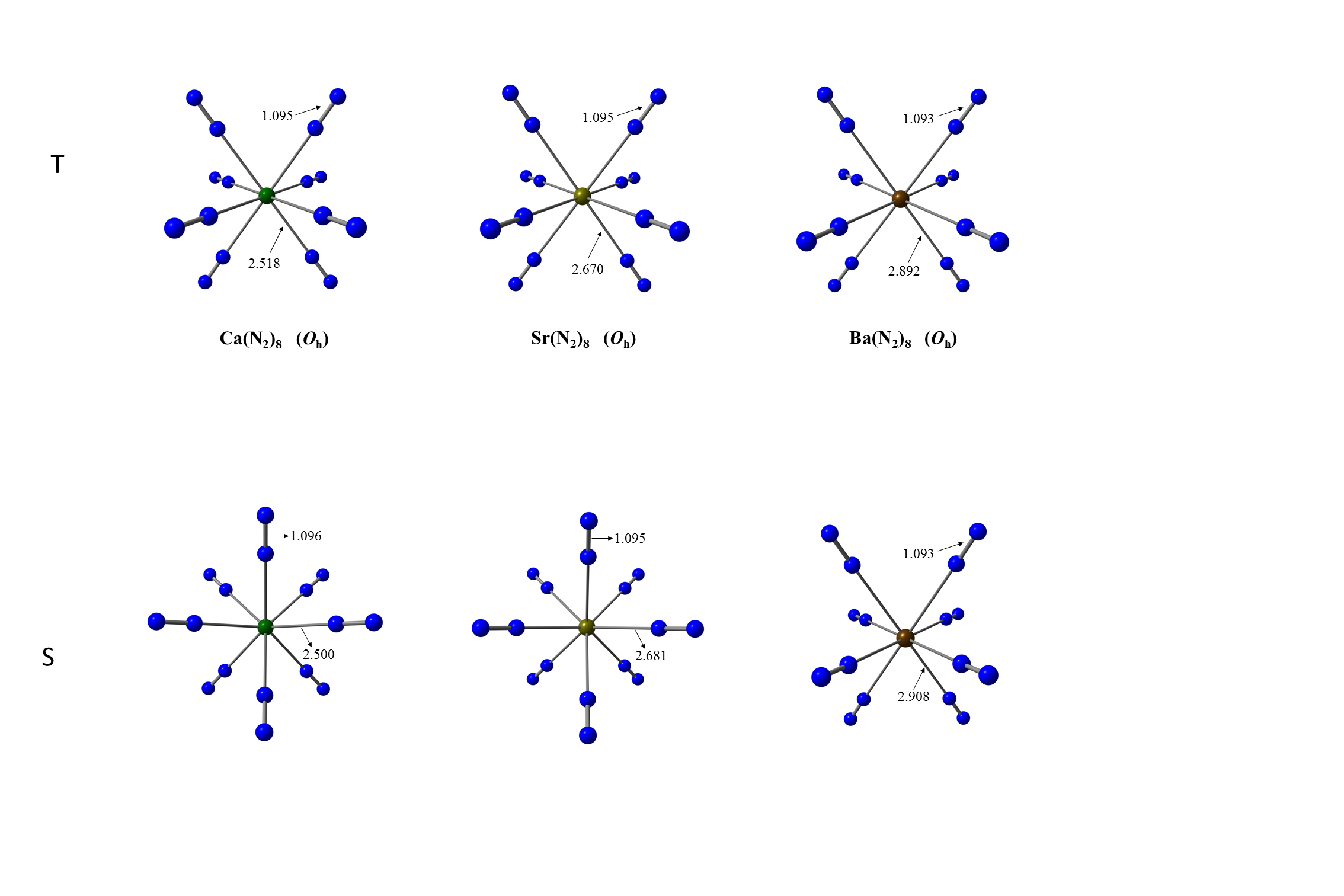  6.2  Ba(N_2_)_8_ (*D*_4_*_h_*, S) |

**Supplementary Figure 8.** The optimized structures of M(N_2_)_8_ complex in triplet and singlet spin states at the M06-2X-D3/def2-TZVPP level. Relative energies are in kcal/mol and bond distances are in Å.

| 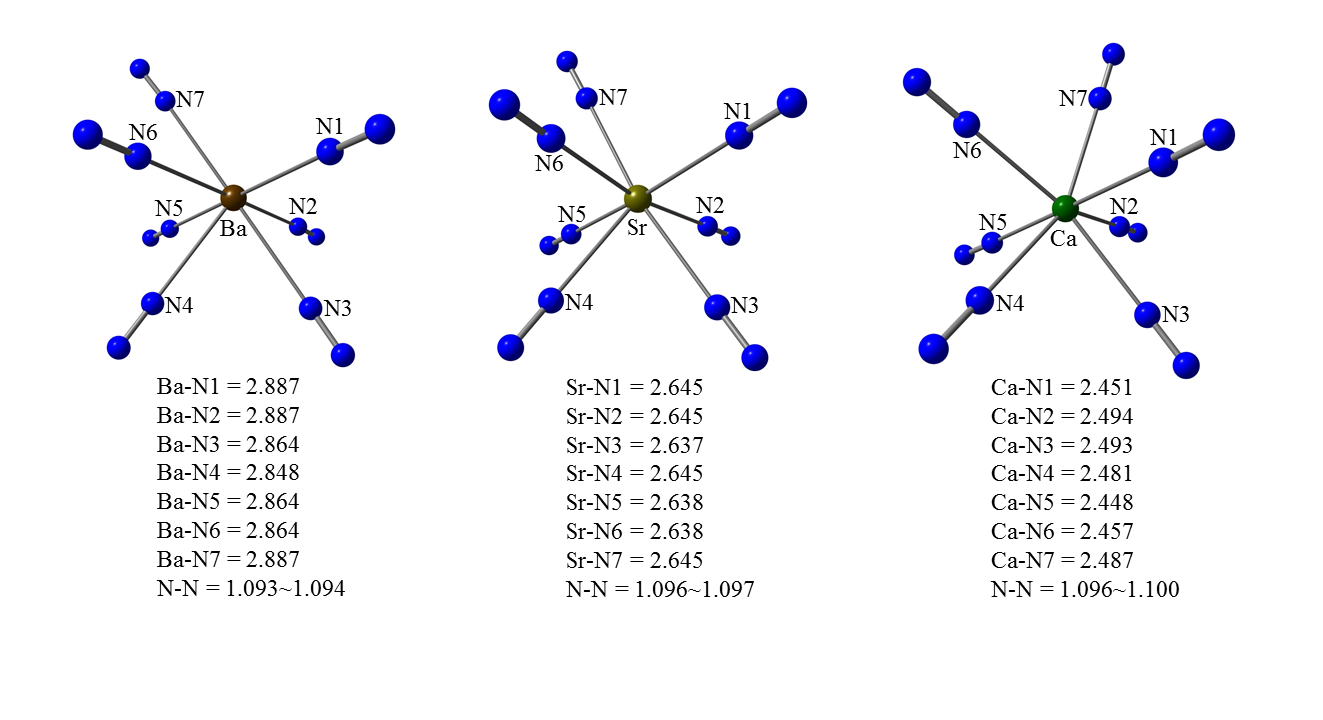  Ca(N_2_)_7_ (*C*_1_, T) | 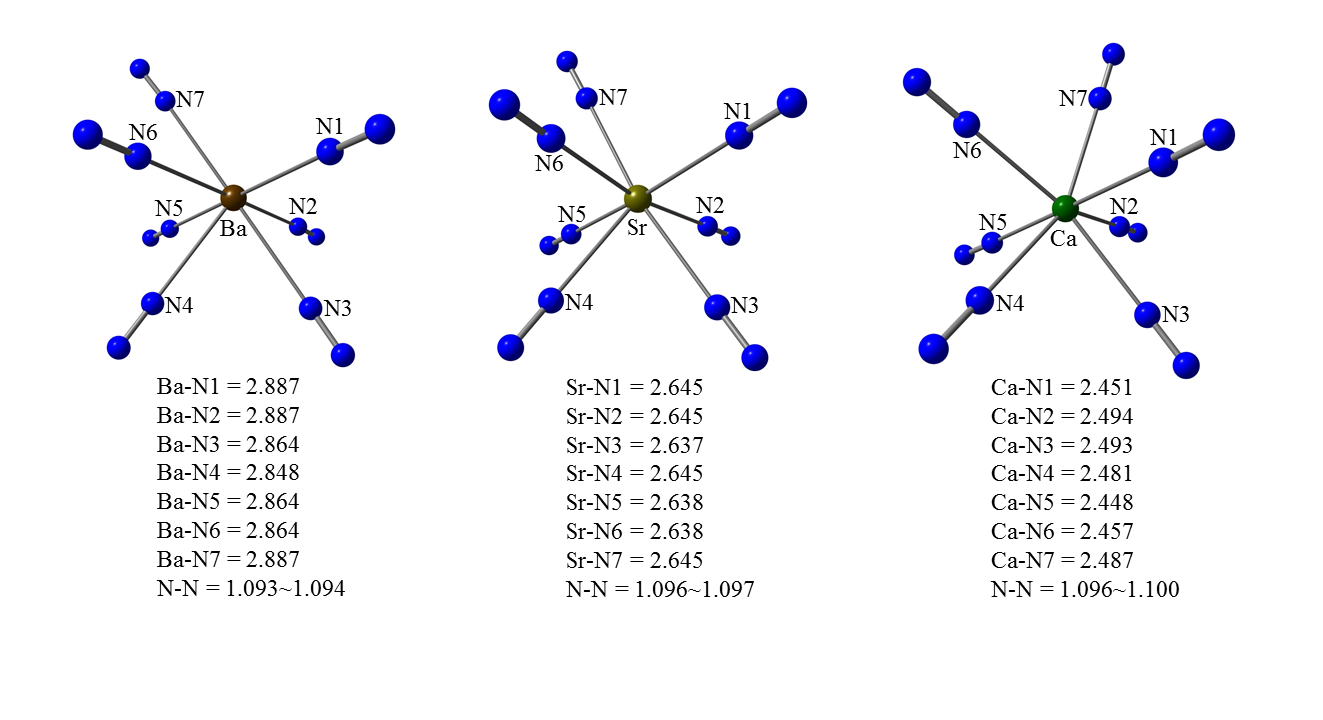  Sr(N_2_)_7_ (*C*_3v_, T) | 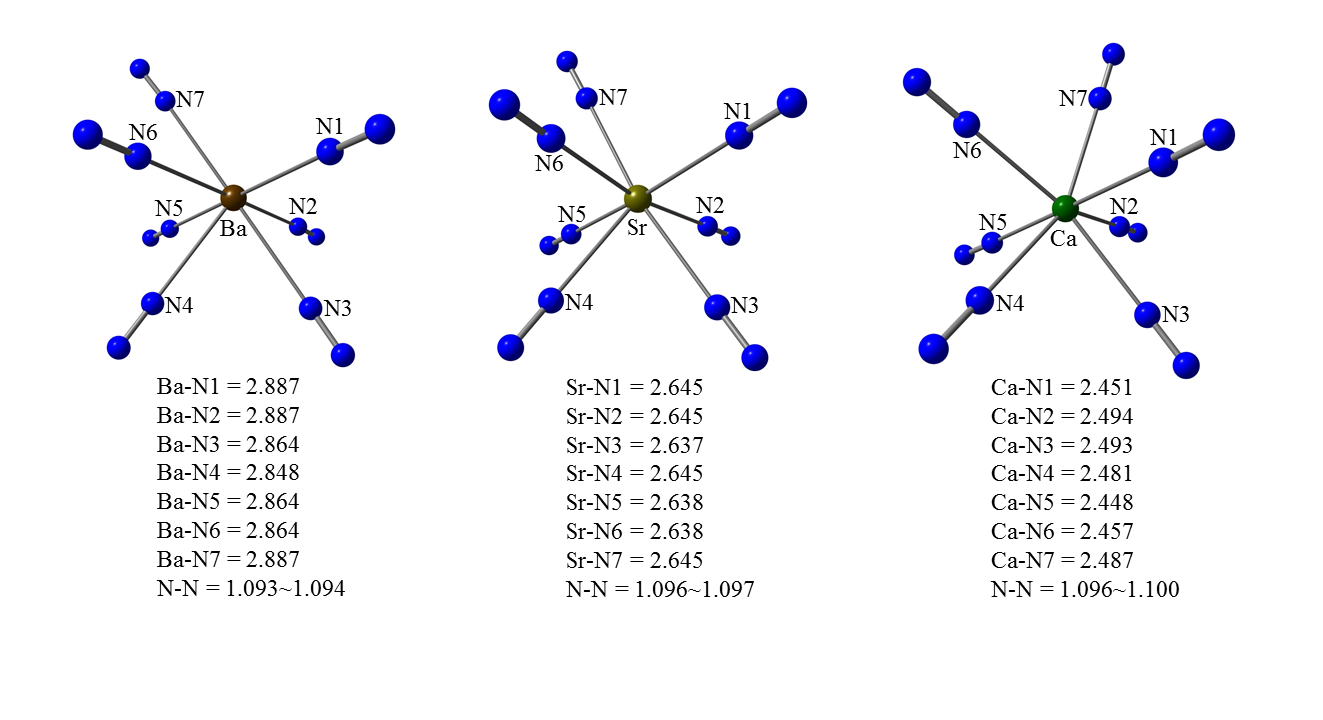  Ba(N_2_)_7_ (*C*_3v_, T) |
| --- | --- | --- |

**Supplementary Figure 9.** The optimized structures of M(N_2_)_7_ complex at the M06-2X-D3/def2-TZVPP level. The bond distances are in Å. The corresponding singlet structures are 5.9 (Ca), 3.5 (Sr) and 2.5 (Ba) kcal/mol higher in energy than the triplet ones.

| 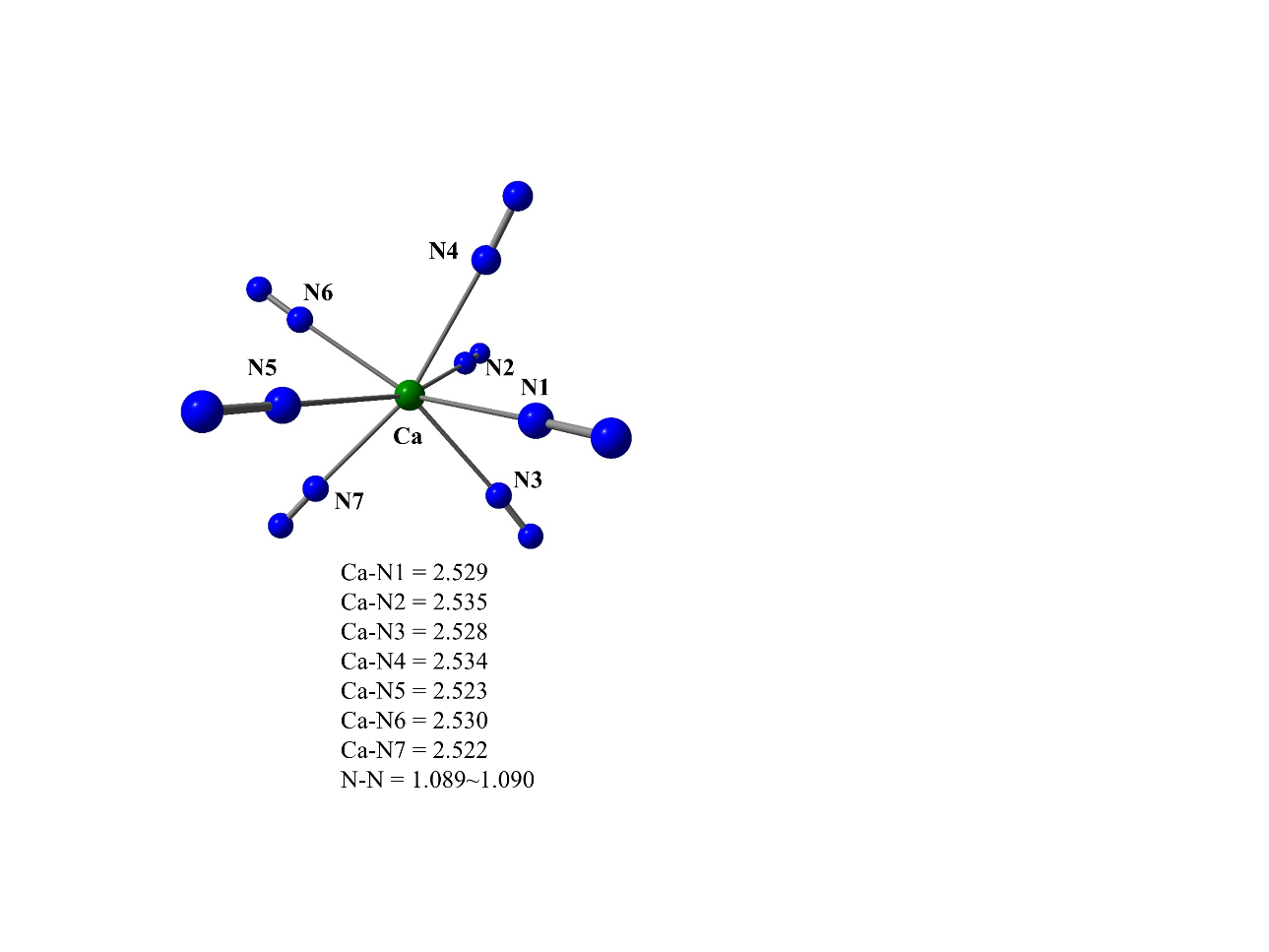  [Ca(N_2_)_7_]^+^ (*C*_1_, D) | 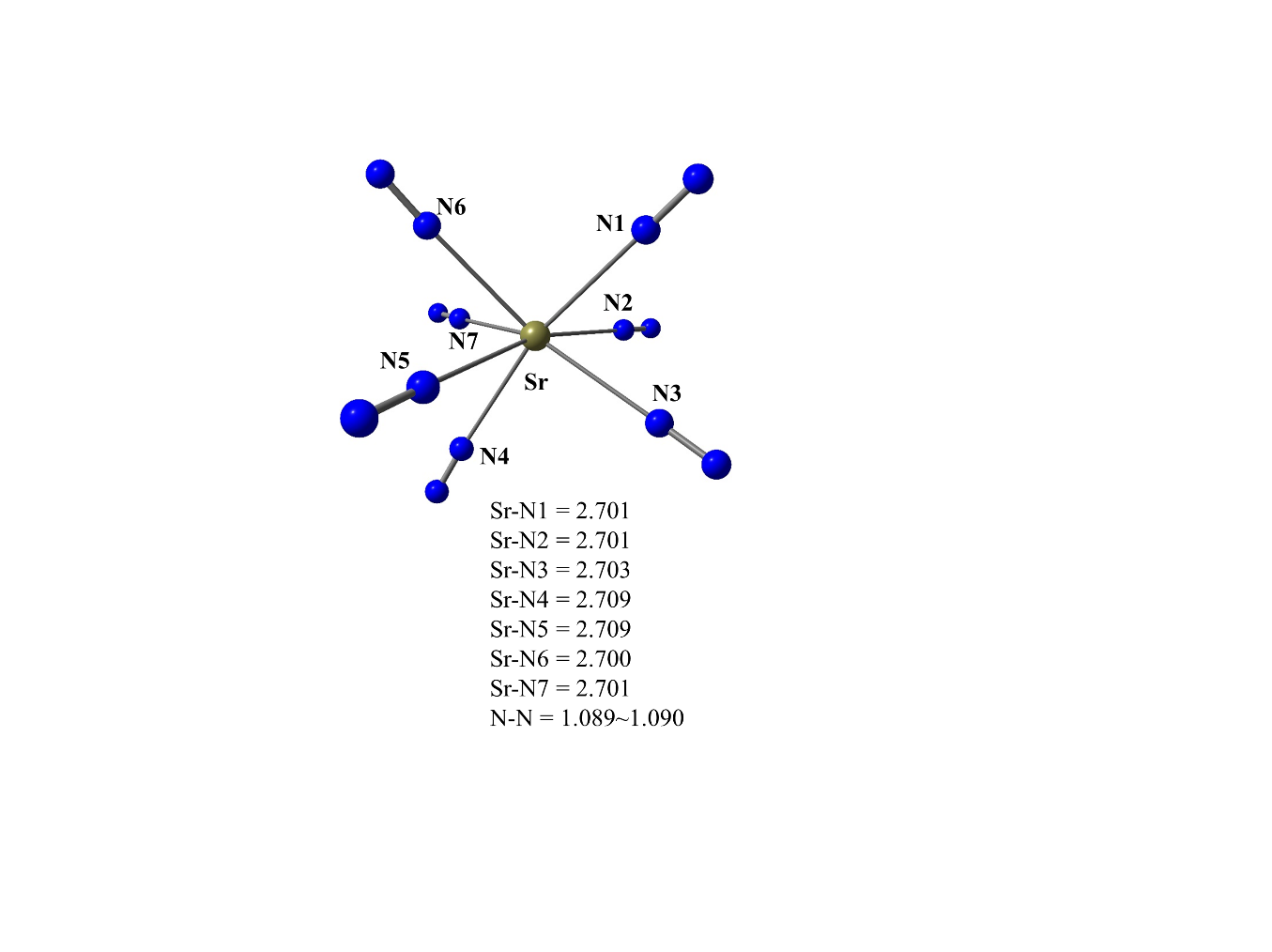  [Sr(N_2_)_7_]^+^ (*C*_1_, D) | 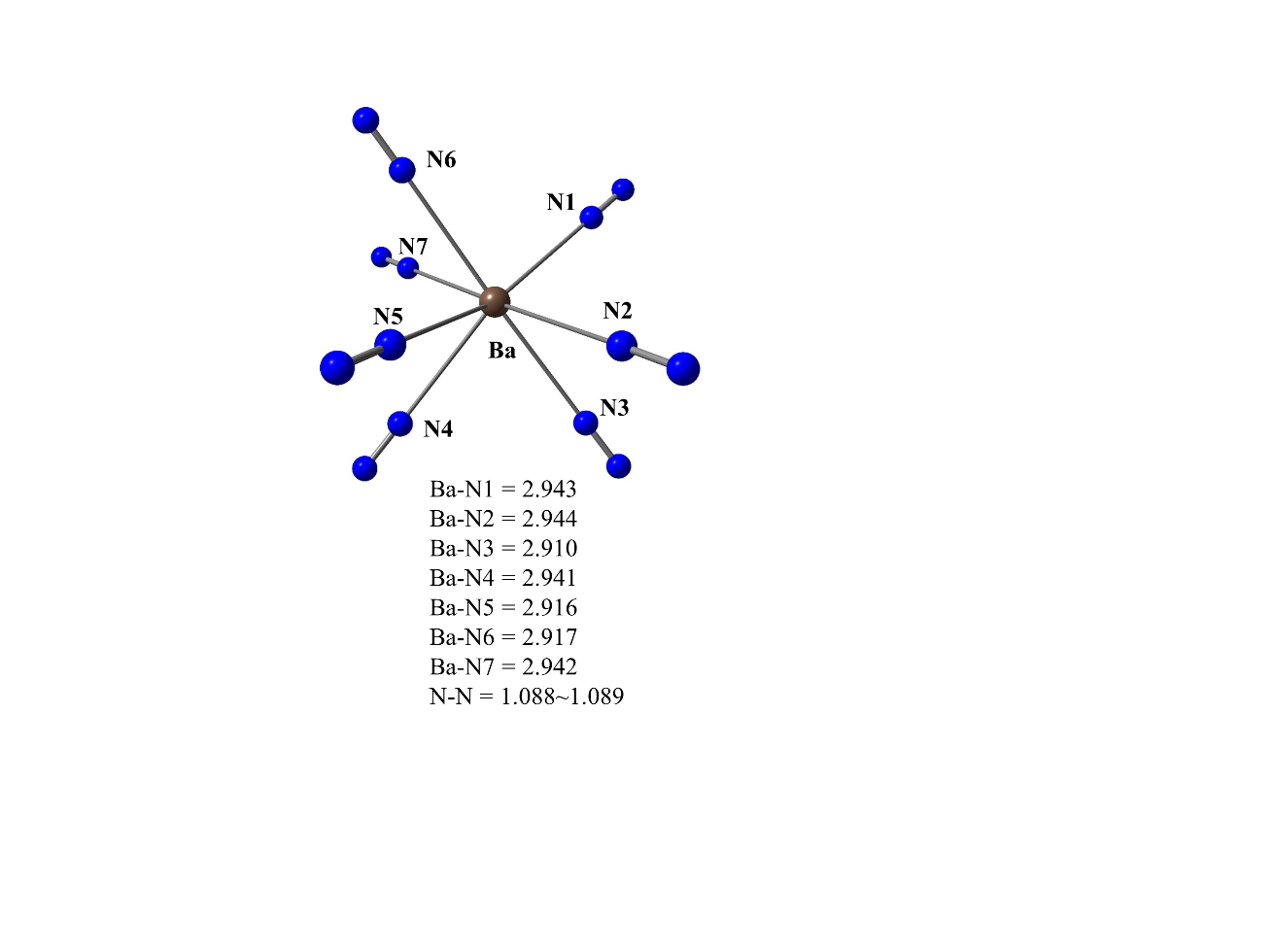  [Ba(N_2_)_7_]^+^ (*C*_1_, D) |
| --- | --- | --- |

**Supplementary Figure 10.** The optimized structures of [M(N_2_)_7_]^+^ complex at the M06-2X-D3/def2-TZVPP level. The bond distances are in Å.

| 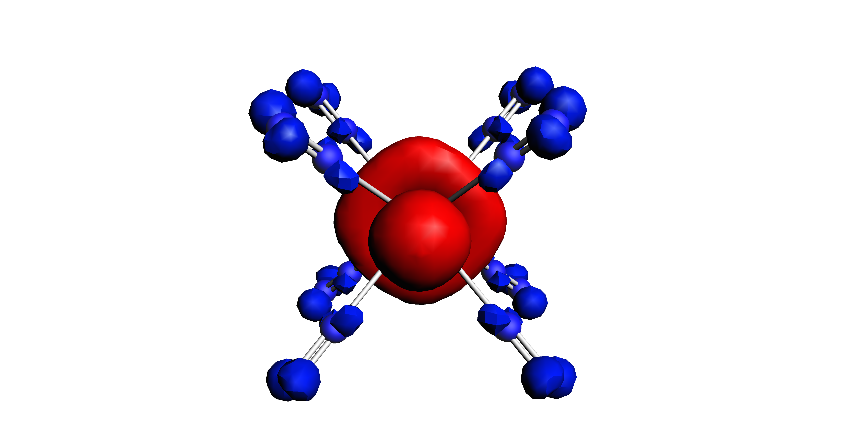  **Δ*E*_orb(1a)_ = -90.3**  **ν_1aα_= 0.73** | 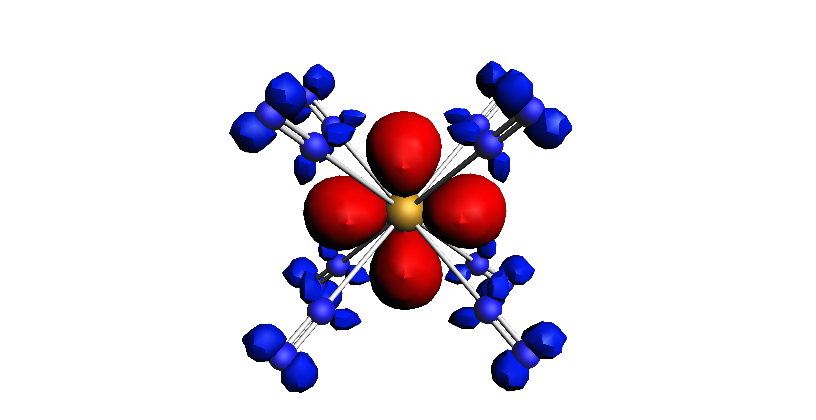  **Δ*E*_orb(1b)_ = -90.3**  **ν_1bα_= 0.73** | 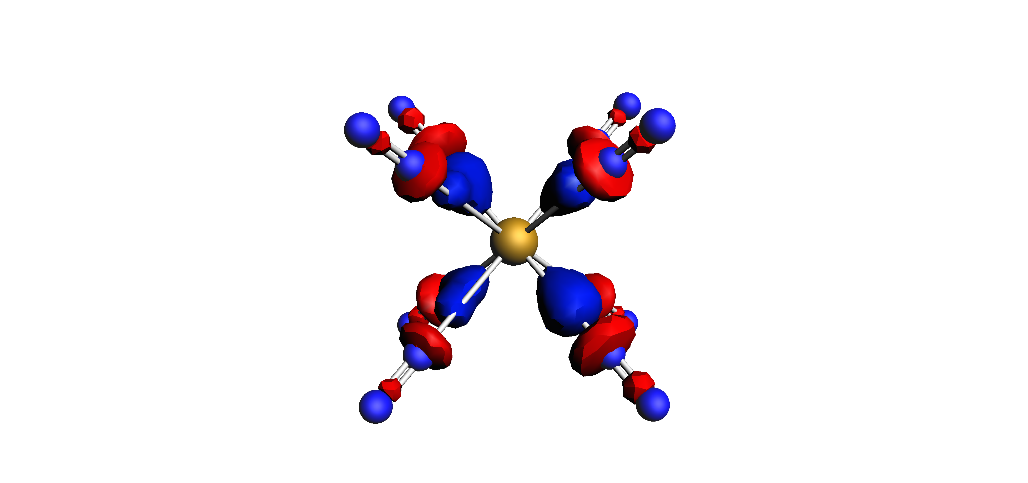  **Δ*E*_orb(2a)_ = -5.8**  **ν_2aα/2aβ_= 0.09/0.08** |
| --- | --- | --- |
| 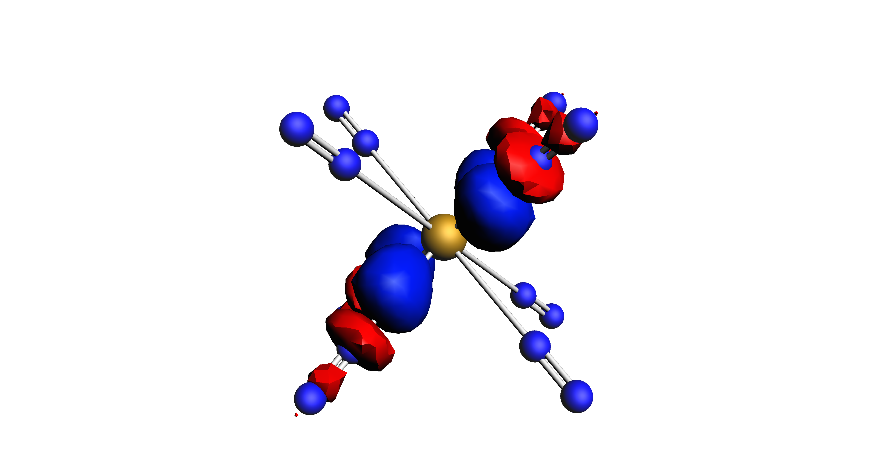  **Δ*E*_orb(2b)_ = -5.8**  **ν_2bα/2bβ_= 0.09/0.08** | 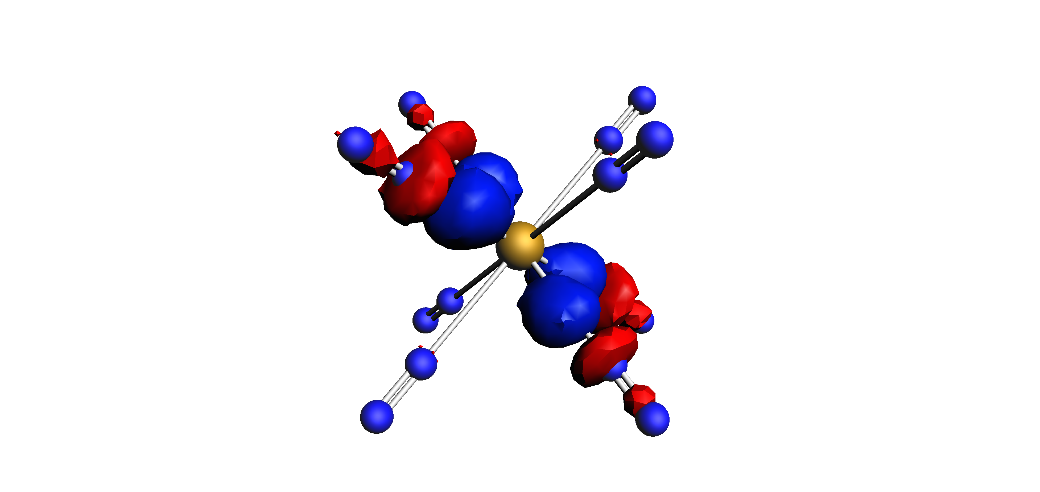  **Δ*E*_orb(2c)_ = -5.8**  **ν_2cα/2cβ_= 0.09/0.08** | 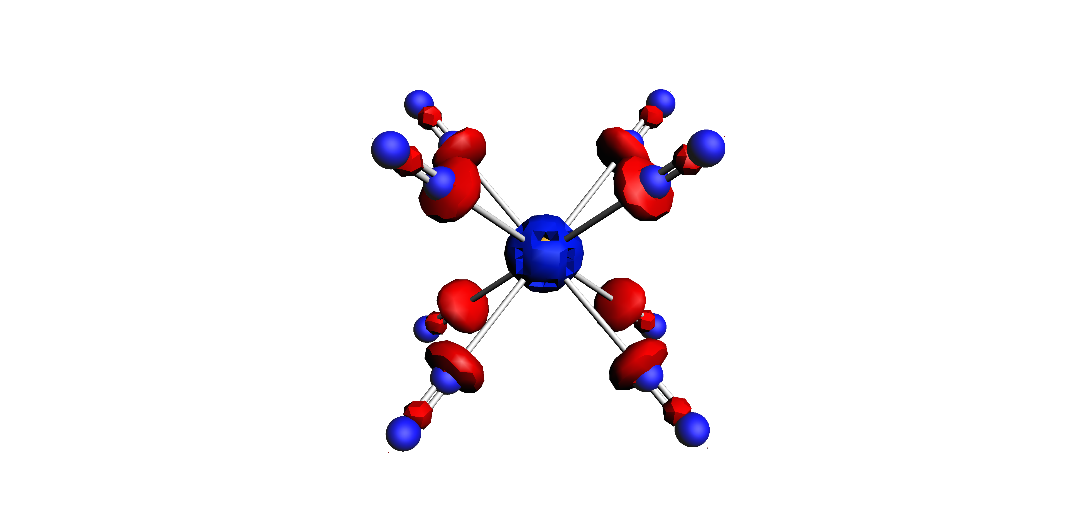  **Δ*E*_orb(3)_ = -3.9**  **ν_3α/3β_= 0.07/0.06** |
| 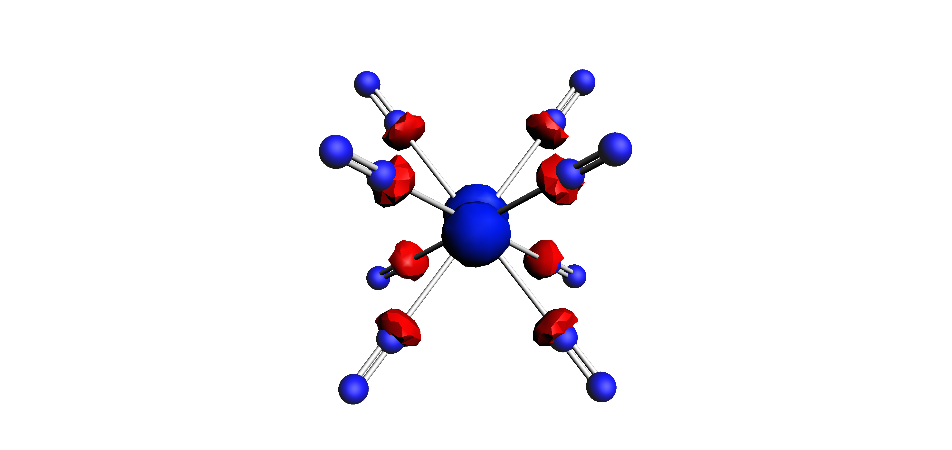  **Δ*E*_orb(4a)_ = -0.9**  **ν_4aα/4aβ_= 0.05** | 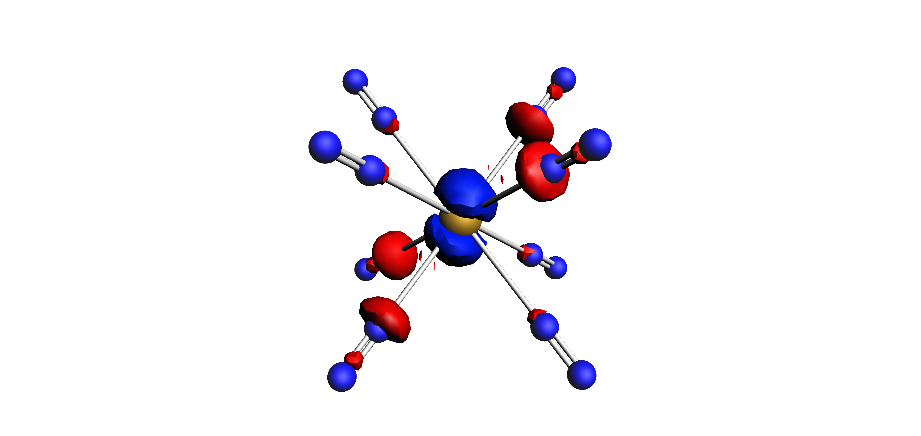  **Δ*E*_orb(4b)_ = -0.9**  **ν_4bα/4bβ_= 0.05** | 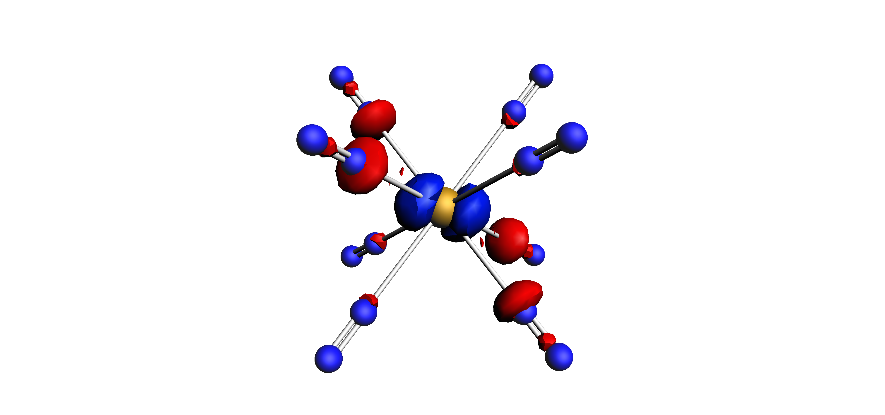  **Δ*E*_orb(4c)_ = -0.9**  **ν_4cα/4cβ_= 0.05** |
|  | 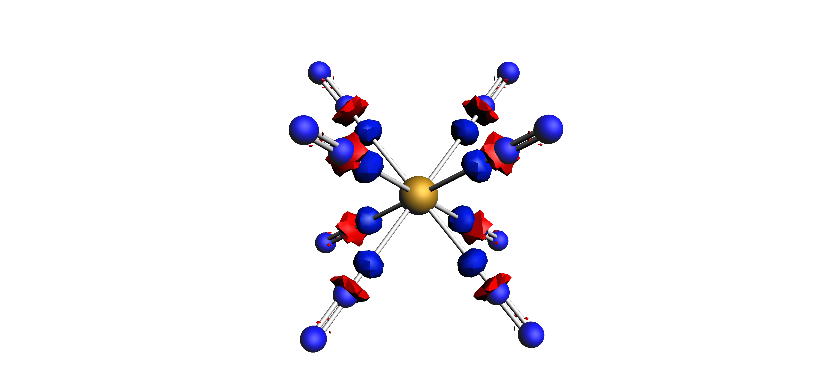  **Δ*E*_orb(5)_ = -1.1**  **ν_5α/5β_= 0.04** |  |

**Supplementary Figure 11.** The plot of deformation densities of triplet *O*_h_ symmetric Sr(N_2_)_8_ complex using neutral partitioning scheme at the M06-2X/TZ2P-ZORA level. Energies are in kcal/mol. The isosurface values are 0.002 for Δ*ρ*_(1)_ and 0.0006 for Δ*ρ*_(2)-(5)_.

| 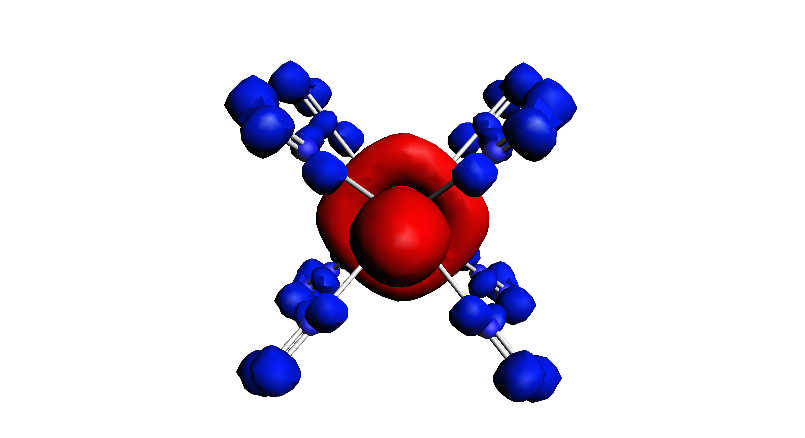  **Δ*E*_orb(1a)_ = -42.7**  **ν_1aα_= 0.57** | 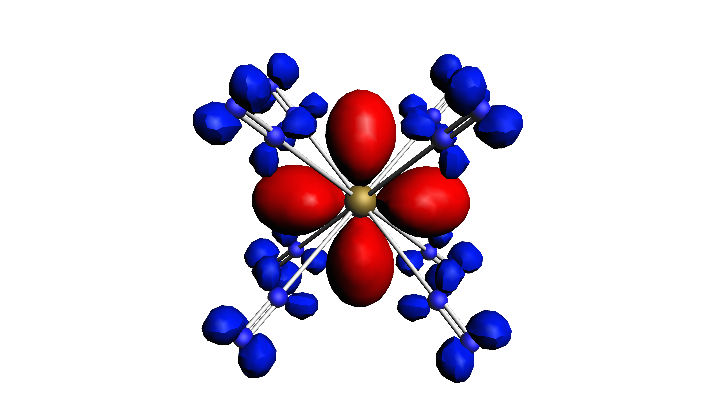  **Δ*E*_orb(1b)_ = -42.3**  **ν_1bα_= 0.56** | 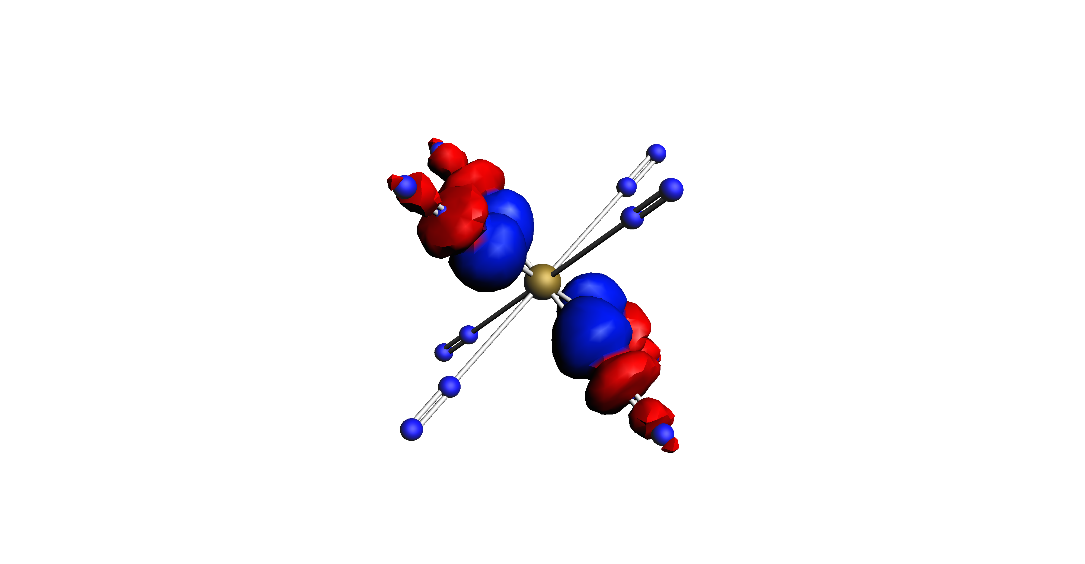  **Δ*E*_orb(2a)_ = -6.0**  **ν_2aα/2aβ_= 0.08/0.07** |
| --- | --- | --- |
| 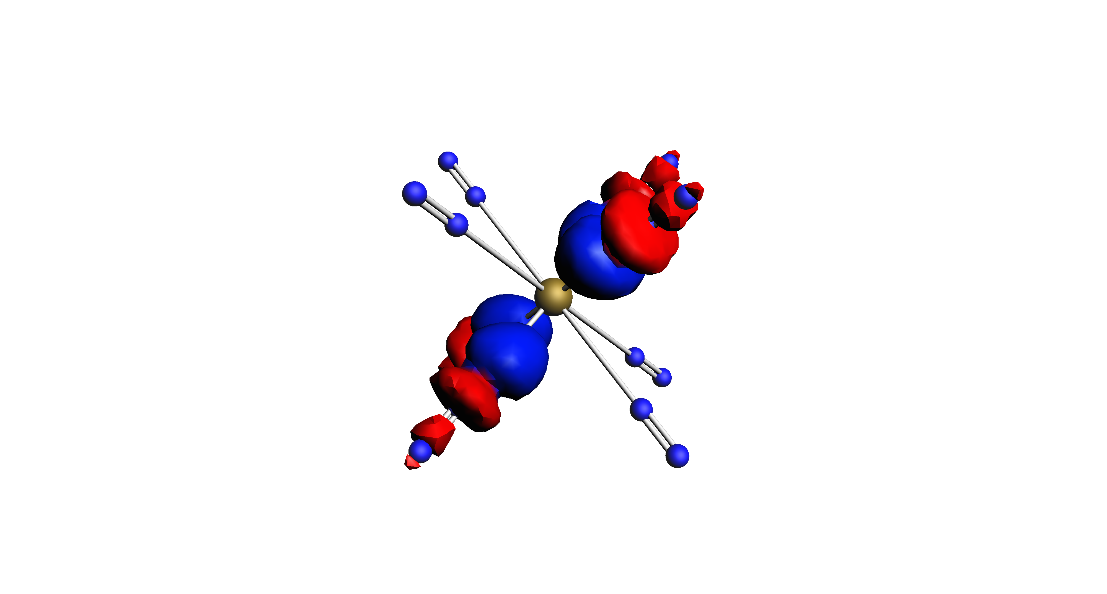  **Δ*E*_orb(2b)_ = -6.0**  **ν_2bα/2bβ_= 0.08/0.07** | 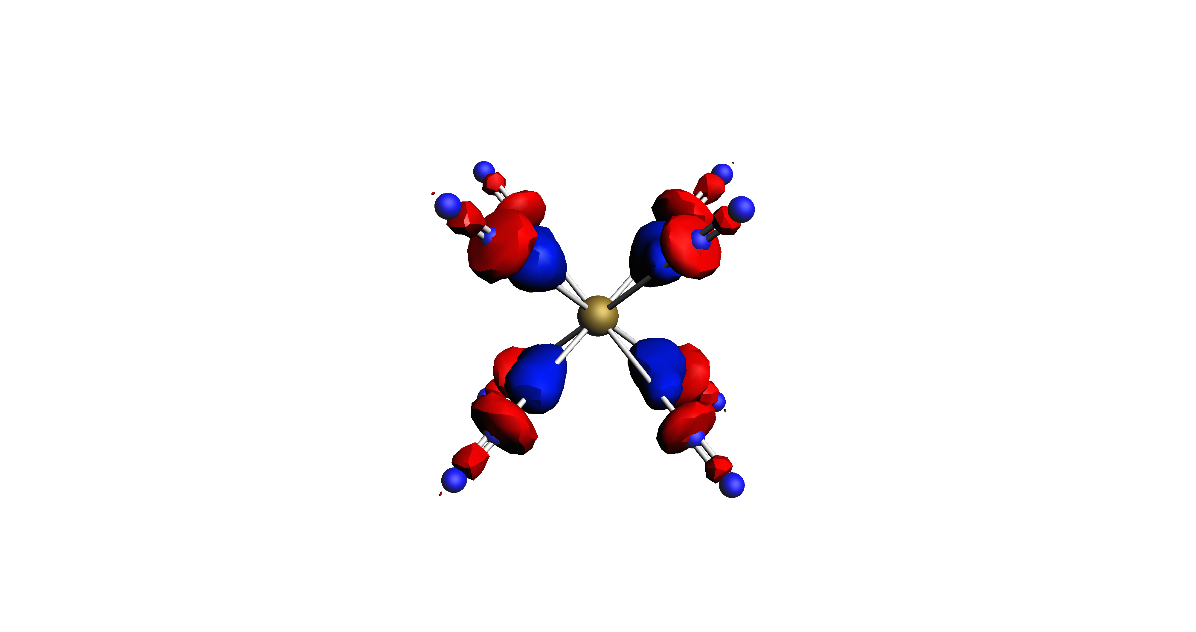  **Δ*E*_orb(2c)_ = -6.0**  **ν_2cα/2cβ_= 0.08/0.07** | 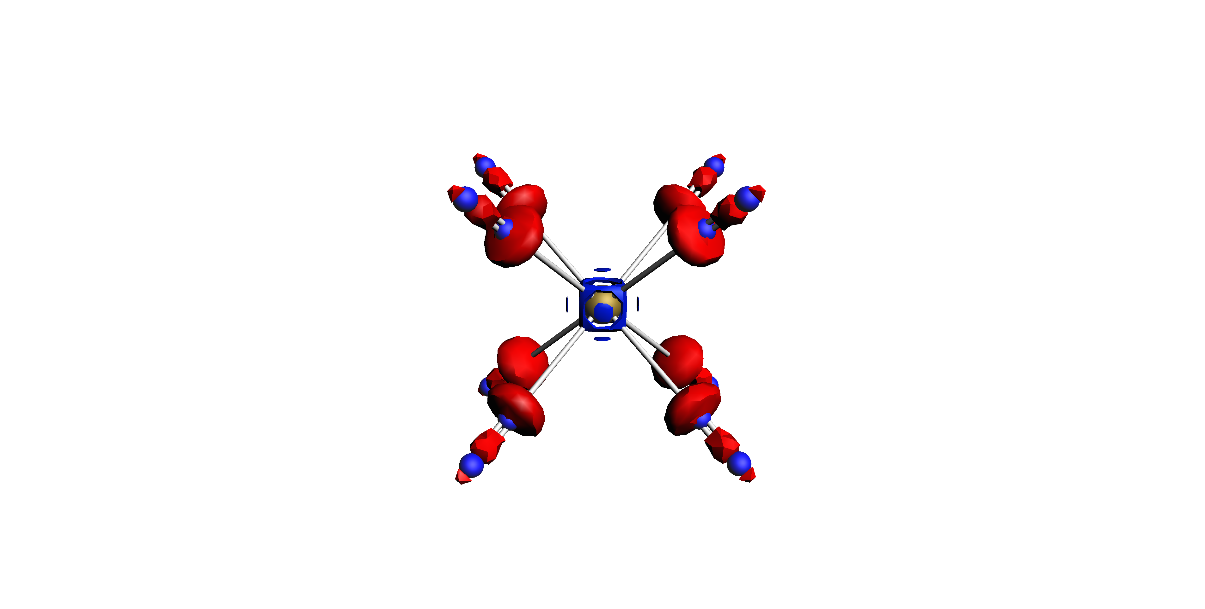  **Δ*E*_orb(3)_ = -3.7**  **ν_3α/3β_= 0.06/0.05** |
| 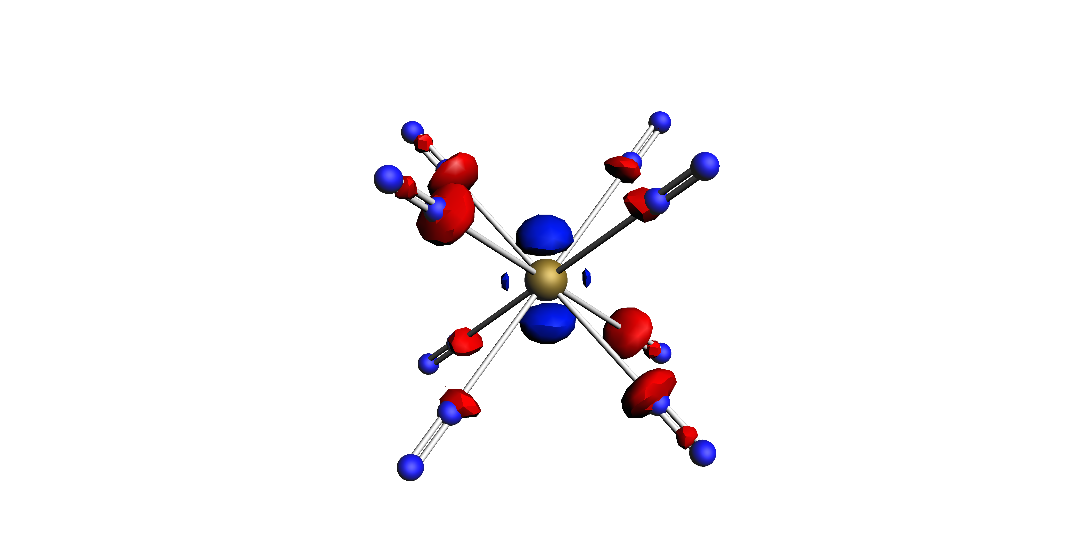  **Δ*E*_orb(4a)_ = -1.4**  **ν_4aα/4aβ_= 0.04** | 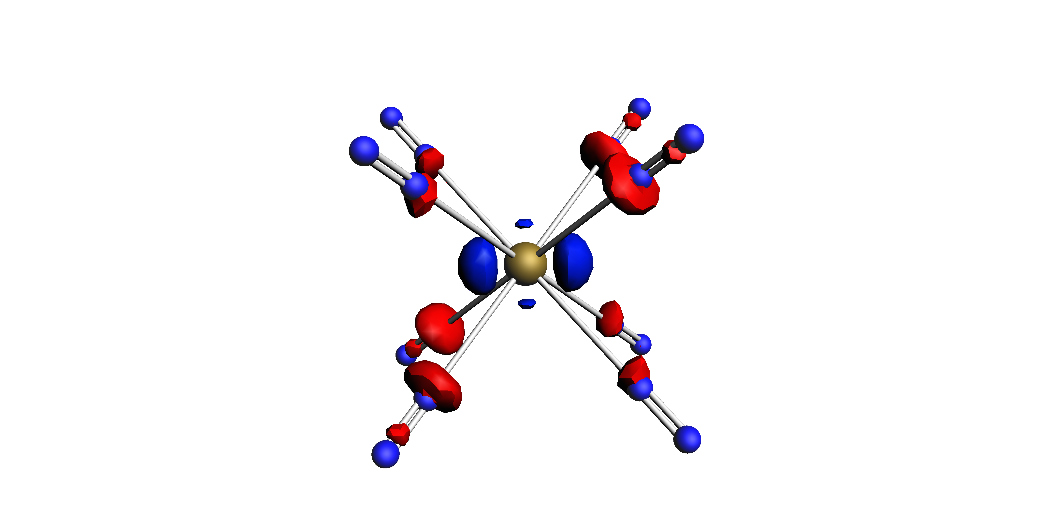  **Δ*E*_orb(4b)_ = -1.4**  **ν_4bα/4bβ_= 0.04** | 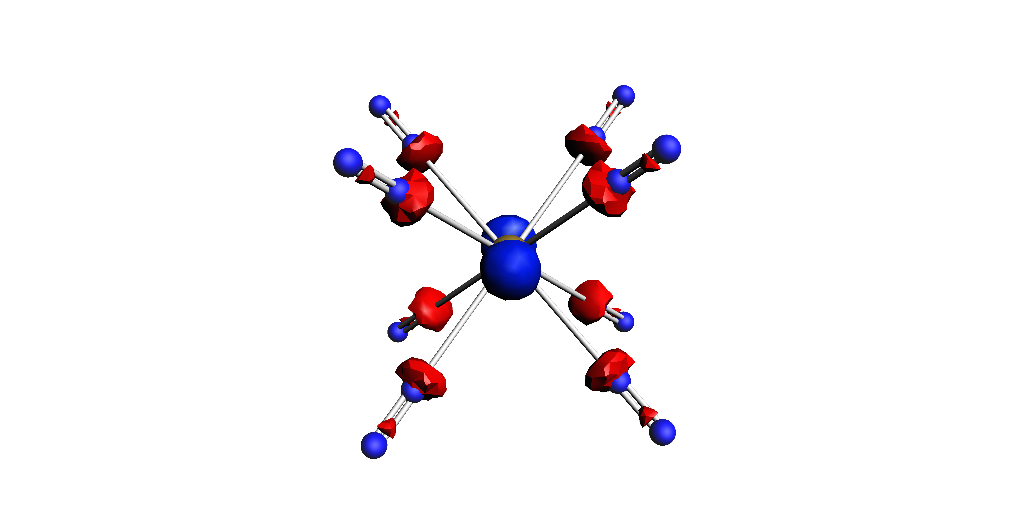  **Δ*E*_orb(4c)_ = -1.4**  **ν_4cα/4cβ_= 0.04** |
|  | 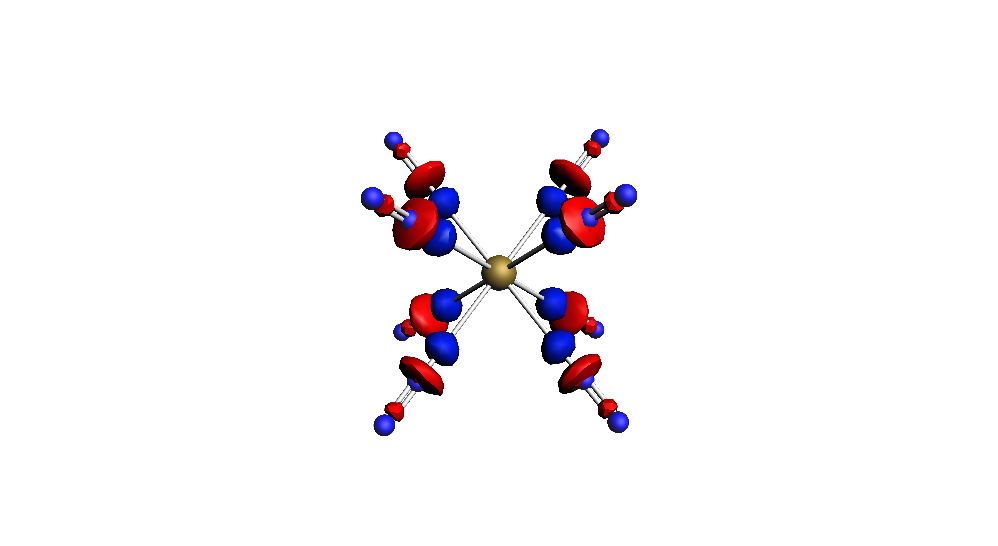  **Δ*E*_orb(5)_ = -1.9**  **ν_5α/5β_= 0.04** |  |

**Supplementary Figure 12.** The plot of deformation densities of triplet *O*_h_ symmetric Ba(N_2_)_8_ complex using neutral partitioning scheme at the M06-2X/TZ2P-ZORA level. Energies are in kcal/mol. The isosurface values are 0.001 for Δ*ρ*_(1)_ and 0.0004 for Δ*ρ*_(2)-(5)_.

**Supplementary Tables**

**Supplementary Table 1.** Calculated unscaled frequencies (**, cm^-1^) and the corresponding IR intensities (I, km/mol) in M(N_2_)_8_ complexes at the M06-2X-D3/def2-TZVPP level.

| Ca(N_2_)_8_ (O_h_, ^3^A_1g_) | | Sr(N_2_)_8_ (O_h_, ^3^A_1g_) | | Ba(N_2_)_8_ (O_h_, ^3^A_1g_) | |
| --- | --- | --- | --- | --- | --- |
| *ν* | *I* | *ν* | *I* | *ν* | *I* |
| 34 (e_u_) | 0 | 32 (e_u_) | 0 | 25 (e_g_) | 0 |
| 61 (t_2g_) | 0 | 47 (t_2g_) | 0 | 28 (t_2u_) | 0 |
| 66 (e_g_) | 0 | 49 (t_2u_) | 0 | 30 (t_2g_) | 0 |
| 66 (t_2u_) | 0 | 51 (e_g_) | 0 | 30 (e_u_) | 0 |
| 74 (t_1u_) | 2 | 54 (t_1u_) | 1 | 34 (t_1u_) | 1 |
| 154 (t_2g_) | 0 | 156 (t_2g_) | 0 | 139 (a_2u_) | 0 |
| 161 (a_2u_) | 0 | 159 (a_2u_) | 0 | 140 (t_2g_) | 0 |
| 195 (a_1g_) | 0 | 182 (a_1g_) | 0 | 151 (a_1g_) | 0 |
| 220 (t_1g_) | 0 | 200 (t_1g_) | 0 | 159 (t_1u_) | 19 |
| 242 (t_2u_) | 0 | 202 (t_1u_) | 32 | 170 (t_1g_) | 0 |
| 243 (t_1u_) | 28 | 210 (e_g_) | 0 | 172 (e_g_) | 0 |
| 249 (e_g_) | 0 | 226 (t_2u_) | 0 | 188 (t_2g_) | 0 |
| 265 (t_2g_) | 0 | 230 (t_2g_) | 0 | 202 (t_2u_) | 0 |
| 266 (e_u_) | 0 | 244 (t_1u_) | 10 | 212 (t_1u_) | 5 |
| 285 (t_1u_) | 41 | 245 (e_u_) | 0 | 212 (e_u_) | 0 |
| 2330 (t_2g_) | 0 | 2341 (t_2g_) | 0 | 2379 (t_2g_) | 0 |
| 2334 (a_2u_) | 0 | 2343 (a_2u_) | 0 | 2379 (a_2u_) | 0 |
| 2341 (t_1u_) | 3603 | 2352 (t_1u_) | 3655 | 2386 (t_1u_) | 2926 |
| 2441 (a_1g_) | 0 | 2438 (a_1g_) | 0 | 2444 (a_1g_) | 0 |

**Supplementary Table 2.** The calculated IR active N-N stretching frequencies υ of M(N_2_)_8_ and [M(N_2_)_8_]^+^ (M = Ca, Sr, Ba) and frequency shifts ∆υ (cm^-1^) at the M06-2X-D3/cc-pCVTZ-pp level.^a^

| Complex | υ(^14^N_2_) | ∆υ^b^ |
| --- | --- | --- |
| Ca(N_2_)_8_ | 2155 | -175 |
| Sr(N_2_)_8_ | 2167 | -163 |
| Ba(N_2_)_8_ | 2197 | -133 |
| [Ca(N_2_)_8_]^+^ | 2243  2246 | -87  -84 |
| [Sr(N_2_)_8_]^+^ | 2250  2252 | -80  -78 |
| [Ba(N_2_)_8_]^+^ | 2275  2276 | -55  -54 |

^a^The calculated values are scaled by 0.923. The scaling factor comes from the ratio of the experimental stretching frequency of 2330 cm^-1^ for N_2_ and the calculated value of 2524 cm^-1^.

^b^Frequency shift relative to free N_2_. The experimental value for N_2_ is 2330 cm^-1^ and the calculated value is 2524 cm^-1^.

**Supplementary Methods**

The geometry optimizations followed by the harmonic frequency calculations for all the systems presented here were carried out at the M06-2X-D3/def2-TZVPP,^[1]^ M06-2X-D3/cc-pCVTZ-PP^[2]^ and B3LYP-D3(BJ)/def2-TZVPPD^[3]^ levels of theory using the Gaussian 16 suit of program.^[4]^ The def2-TZVPP and def2-TZVPPD basis set have scalar-relativistic effective core potentials (ECPs) for 28 and 46 core electrons for Sr and Ba, respectively.^[5]^ The cc-pCVTZ-PP basis set uses fully relativistic ECPs replacing 10, 28 and 46 core electrons of Ca, Sr and Ba, respectively.^[2]^ Superfine integration grid is considered for all cases.

The energy decomposition analysis (EDA)^[6]^ in combination with natural orbital for chemical valence (NOCV)^[7]^ method was performed at the M06-2X/TZ2P-ZORA//M06-2X-D3/def2-TZVPP level^[8]^ using the ADF (2017.101) program package.^[9]^ The zeroth-order regular approximation (ZORA) was used to include scalar relativistic effects for the metals.^[10]^ All electrons were considered in the computations.

In the EDA method, the interaction energy (Δ*Ε*_int_) between two prepared fragments is divided into three energy terms, viz., the electrostatic interaction energy (Δ*E*_elstat_), which represents the quasiclassical electrostatic interaction between the unperturbed charge distributions of the prepared atoms, the Pauli repulsion (Δ*E*_Pauli_), which is the energy change associated with the transformation from the superposition of the unperturbed electron densities of the isolated fragments to the wavefunction that properly obeys the Pauli principle through explicit antisymmetrization and renormalization of the product wavefunction, and the orbital interaction energy (Δ*E*_orb_), which is originated from the mixing of orbitals, charge transfer and polarization between the isolated fragments. Therefore, the interaction energy (Δ*Ε*_int_) between two fragments can be defined as:

Δ*Ε*_int_ = Δ*E*_elstat_ + Δ*E*_Pauli_ + Δ*E*_orb_ (1)

Since metahybrid M06-2X functional is considered in the present study, the so-called transition state procedure uses an approximate Fock operator in the computations, and, therefore, it adds additional metahybrid correction, Δ*Ε*_hybrid_, towards the Δ*E*_orb_ term.

The EDA-NOCV calculation combines charge and energy decomposition schemes to divide the deformation density, Δ*ρ*(**r**), associated with the bond formation into different components (σ, π, δ) of a chemical bond. From the mathematical point of view, each NOCV, 𝜓_i_ is defined as an eigenvector of the deformation density matrix in the basis of fragment orbitals.

Δ*P*ψ_i_ = *ν*_i_ψ_I_ (2)

In EDA-NOCV, Δ*E*_orb_ is given by the following equation

$\Delta E_{orb}=\sum_{k} \Delta E_{k}^{orb}=\sum_{k=1}^{N/2} \nu_{k}[-F_{-k}^{TS}+F_{k}^{TS}]$ (3)

where, $-F_{-k}^{TS}$and $F_{k}^{TS}$ are diagonal Kohn-Sham matrix elements corresponding to NOCVs with the eigenvalues –*ν*_k_ and *ν*_k_, respectively. The $\Delta E_{k}^{orb}$terms are assigned to a particular type of bond by visual inspection of the shape of the deformation density, ∆*ρ*_k_. The EDA-NOCV scheme thus provides both qualitative (∆*ρ*_orb_) and quantitative (∆*E*_orb_) information about the strength of orbital interactions in chemical bonds. More details about EDA-NOCV and its application can be found in recent reviews.^[11]^

**References**

[1] a) Y. Zhao, D. G. Truhlar, *Theor Chem Acc*. **2006**, *120*, 215–241; b) S. Grimme, J. Antony, S. Ehrlich, H. Krieg, *J. Chem. Phys*. **2010**, *132*, 154104; c) F. Weigend, R. Ahlrichs, *Phys. Chem. Chem. Phys*. **2005**, *7*, 3297-3305.

[2] a) H. Li, H. Feng, W. Sun, Y. Zhang, Q. Fan, K. A. Peterson, Y., Xie, H. F. Schaefer III, *Mol. Phys*. **2013**, *111*, 2292; b) I. S. Lim, H. Stoll, P. Schwerdtfeger, *J. Chem. Phys*. **2006**, *124*, 034107.

[3] a) A. D. Becke, *Phys. Rev. A* **1988**, *38*, 3098 ; b) C. Lee, W. Yang, R. G. Parr, *Phys. Rev. B* **1988**, *37*, 785-789.

[4] Gaussian 16, Revision A.03, M. J. Frisch, G. W. Trucks, H. B. Schlegel, G. E. Scuseria, M. A. Robb, J. R. Cheeseman, G. Scalmani, V. Barone, G. A. Petersson, H. Nakatsuji, X. Li, M. Caricato, A. V. Marenich, J. Bloino, B. G. Janesko, R. Gomperts, B. Mennucci, H. P. Hratchian, J. V. Ortiz, A. F. Izmaylov, J. L. Sonnenberg, D. Williams-Young, F. Ding, F. Lipparini, F. Egidi, J. Goings, B. Peng, A. Petrone, T. Henderson, D. Ranasinghe, V. G. Zakrzewski, J. Gao, N. Rega, G. Zheng, W. Liang, M. Hada, M. Ehara, K. Toyota, R. Fukuda, J. Hasegawa, M. Ishida, T. Nakajima, Y. Honda, O. Kitao, H. Nakai, T. Vreven, K. Throssell, J. A. Montgomery, Jr., J. E. Peralta, F. Ogliaro, M. J. Bearpark, J. J. Heyd, E. N. Brothers, K. N. Kudin, V. N. Staroverov, T. A. Keith, R. Kobayashi, J. Normand, K. Raghavachari, A. P. Rendell, J. C. Burant, S. S. Iyengar, J. Tomasi, M. Cossi, J. M. Millam, M. Klene, C. Adamo, R. Cammi, J. W. Ochterski, R. L. Martin, K. Morokuma, O. Farkas, J. B. Foresman, D. J. Fox, Gaussian, Inc., Wallingford CT, **2016**.

[5] M. Kaupp, P. V. Schleyer, H. Stoll, H. Preuss, *J. Chem. Phys*. **1991**, *94*, 1360-1366.

[6] T. Ziegler, A. Rauk, *Theor. Chim. Acta* **1977**, *46*, 1-10.

[7] a) M. Mitoraj, A. Michalak, *Organometallics* **2007**, *26*, 6576-6580; b) A. Michalak, M. Mitoraj, T. Ziegler, *J. Phys. Chem. A* **2008,** *112*, 1933.

[8] E. van Lenthe, E. J. Baerends, *J. Comput. Chem*. **2003**, *24*, 1142-1156.

[9] a) ADF2017, SCM, Theoretical Chemistry, Vrije Universiteit, Amsterdam, The Netherlands, [http://www.scm.com](http://www.scm.com/); b) G. te Velde, F. M. Bickelhaupt, E. J. Baerends, C. F. Guerra, S. J. A. Van Gisbergen, J. G. Snijders, T. Ziegler, *J. Comput. Chem*. **2001**, *22*, 931-967.

[10] a) Ch. Chang, M. Pelissier, Ph. Durand, *Phys. Scr*. **1986**, *34*, 394-401; b) J.-L. Heully, I. Lindgren, E. Lindroth, S. Lundqvist, A.-M. Martensson-Pendrill, *J. Phys. B* **1986**, *19*, 2799-2805; c) E. van Lenthe, E. J. Baerends, J. G. Snijders, *J. Chem. Phys*. **1993**, 99, 4597-4603.

[11] a) G. Frenking, F. M. Bickelhaupt, The EDA Perspective of Chemical Bonding. In *The Chemical Bond 1. Fundamental Aspects of Chemical Bonding*, G. Frenking, S. Shaik, Eds. Wiley-VCH: Weinheim, 2014, pp 121- 158; b) L. Zhao, M. Hermann, N. Holzmann, G. Frenking, *Coord. Chem. Rev.* **2017**, *344*, 163-204; c) G. Frenking, M. Hermann, D. M. Andrada, N. Holzmann, *Chem. Soc. Rev*. **2016**, *45*, 1129-1144.
