# Supplementary material for: Octa-coordinated alkaline earth metal–dinitrogen complexes M(N2)8 (M=Ca, Sr, Ba)
Source: Nat Commun. 2019 Jul 29;10:3375. doi: 10.1038/s41467-019-11323-5 (PMC6662891; doi:10.1038/s41467-019-11323-5)
Supplement: Supplementary file 2 — Description of Additional Supplementary Files [file 41467_2019_11323_MOESM2_ESM.docx]

Description of Additional Supplementary Files

**Supplementary Data 1:** Coordinates and energies of the calculated molecules at the M06-2X-D3/def2-TZVPP level.
