# Supplementary material for: Octa-coordinated alkaline earth metal–dinitrogen complexes M(N2)8 (M=Ca, Sr, Ba)
Source: Nat Commun. 2019 Jul 29;10:3375. doi: 10.1038/s41467-019-11323-5 (PMC6662891; doi:10.1038/s41467-019-11323-5)
Supplement: Supplementary file 3 — Supplementary Data 1 [file 41467_2019_11323_MOESM3_ESM.pdf]

Supplementary Data 1. Coordinates and energies of the calculated molecules at the M06-2X-D3/def2-TZVPP level.

$Ca(N_2)_8$ (O_h, T)
E=-1553.88300103 au
0 3
Ca 0.000000 0.000000 0.000000
N 1.453962 1.453962 1.453962
N -1.453962 1.453962 -1.453962
N -1.453962 -1.453962 -1.453962
N -1.453962 1.453962 1.453962
N 1.453962 -1.453962 1.453962
N 1.453962 1.453962 -1.453962
N -1.453962 -1.453962 1.453962
N 1.453962 -1.453962 -1.453962
N 2.086427 -2.086427 -2.086427
N 2.086427 -2.086427 2.086427
N -2.086427 -2.086427 2.086427
N -2.086427 -2.086427 -2.086427
N -2.086427 2.086427 -2.086427
N 2.086427 2.086427 -2.086427
N 2.086427 2.086427 2.086427
N -2.086427 2.086427 2.086427

$Sr(N_2)_8$ (O_h, T)
E=-906.946153910 au
0 3
Sr 0.000000 0.000000 0.000000
N 1.541669 1.541669 1.541669
N -1.541669 1.541669 -1.541669
N -1.541669 -1.541669 -1.541669
N -1.541669 1.541669 1.541669
N 1.541669 -1.541669 1.541669
N 1.541669 1.541669 -1.541669
N -1.541669 -1.541669 1.541669
N 1.541669 -1.541669 -1.541669
N 2.173792 -2.173792 -2.173792
N 2.173792 -2.173792 2.173792
N -2.173792 -2.173792 2.173792
N -2.173792 -2.173792 -2.173792
N -2.173792 2.173792 -2.173792
N 2.173792 2.173792 -2.173792
N 2.173792 2.173792 2.173792
N -2.173792 2.173792 2.173792

$Ba(N_2)_8$ (O_h, T)
E=-901.725009405 au
0 3
Ba 0.000000 0.000000 0.000000

N	1.669419	1.669419	1.669419
N	-1.669419	1.669419	-1.669419
N	-1.669419	-1.669419	-1.669419
N	-1.669419	1.669419	1.669419
N	1.669419	-1.669419	1.669419
N	1.669419	1.669419	-1.669419
N	-1.669419	-1.669419	1.669419
N	1.669419	-1.669419	-1.669419
N	2.300462	-2.300462	-2.300462
N	2.300462	-2.300462	2.300462
N	-2.300462	-2.300462	2.300462
N	-2.300462	-2.300462	-2.300462
N	-2.300462	2.300462	-2.300462
N	2.300462	2.300462	-2.300462
N	2.300462	2.300462	2.300462
N	-2.300462	2.300462	2.300462

$[Ca(N_2)_8]^+$ (D_{4d}, D)

E = -1553.7186052 au

Ca	0.000000000	0.000000000	0.000000000
N	1.480161000	-1.480161000	-1.454515000
N	-1.480161000	-1.480161000	-1.454515000
N	-1.480161000	1.480161000	-1.454515000
N	0.000000000	2.093264000	1.454515000
N	2.093264000	0.000000000	1.454515000
N	0.000000000	-2.093264000	1.454515000
N	1.480161000	1.480161000	-1.454515000
N	-2.093264000	0.000000000	1.454515000
N	2.128622000	2.128622000	-2.043497000
N	0.000000000	3.010326000	2.043497000
N	-2.128622000	2.128622000	-2.043497000
N	-2.128622000	-2.128622000	-2.043497000
N	0.000000000	-3.010326000	2.043497000
N	-3.010326000	0.000000000	2.043497000
N	3.010326000	0.000000000	2.043497000
N	2.128622000	-2.128622000	-2.043497000

$[Sr(N_2)_8]^+$ (D_{4d}, D)

E = -906.7858101 au

Sr	0.000000000	0.000000000	0.000000000
N	0.000000000	2.157245000	1.654657000
N	-2.157245000	0.000000000	1.654657000
N	0.000000000	-2.157245000	1.654657000
N	1.525403000	-1.525403000	-1.654657000
N	1.525403000	1.525403000	-1.654657000
N	-1.525403000	1.525403000	-1.654657000
N	2.157245000	0.000000000	1.654657000
N	-1.525403000	-1.525403000	-1.654657000
N	3.040866000	0.000000000	2.291058000
N	2.150217000	-2.150217000	-2.291058000
N	0.000000000	-3.040866000	2.291058000
N	-3.040866000	0.000000000	2.291058000

N	-2.150217000	2.150217000	-2.291058000
N	-2.150217000	-2.150217000	-2.291058000
N	2.150217000	2.150217000	-2.291058000
N	0.000000000	3.040866000	2.291058000
$[Ba(N_2)_8]^+$ (D_{4h}, D)			
E = -901.5679065 au			
Ba	0.000000000	0.000000000	0.000000000
N	0.000000000	2.260760000	1.891070000
N	0.000000000	2.260760000	-1.891070000
N	2.260760000	0.000000000	-1.891070000
N	2.260760000	0.000000000	1.891070000
N	-2.260760000	0.000000000	1.891070000
N	0.000000000	-2.260760000	1.891070000
N	0.000000000	-2.260760000	-1.891070000
N	-2.260760000	0.000000000	-1.891070000
N	3.096190000	0.000000000	-2.588248000
N	0.000000000	3.096190000	-2.588248000
N	0.000000000	3.096190000	2.588248000
N	-3.096190000	0.000000000	2.588248000
N	0.000000000	-3.096190000	2.588248000
N	3.096190000	0.000000000	2.588248000
N	-3.096190000	0.000000000	-2.588248000
N	0.000000000	-3.096190000	-2.588248000
$Ba(N_2)_7$ (C_{3v}, T)			
E=-792.174101435 au			
0 3			
Ba	0.000000	0.000000	0.318687
N	0.000000	-2.705002	1.326684
N	-2.342600	1.352501	1.326684
N	2.342600	1.352501	1.326684
N	3.203735	-1.849677	-1.090590
N	0.000000	3.699355	-1.090590
N	-3.203735	-1.849677	-1.090590
N	0.000000	2.677389	-0.698851
N	-2.318687	-1.338694	-0.698851
N	2.318687	-1.338694	-0.698851
N	0.000000	0.000000	-3.623858
N	-3.244168	1.873021	1.662827
N	0.000000	-3.746043	1.662827
N	3.244168	1.873021	1.662827
N	0.000000	0.000000	-2.529416
$Sr(N_2)_7$ (C_{3v}, T)			
E=-797.393310103 au			
0 3			
Sr	0.000000	0.000000	0.188941
N	0.000000	2.496671	-0.662561
N	2.162181	-1.248336	-0.662561
N	-2.162181	-1.248336	-0.662561
N	0.000000	3.539447	-1.002404
N	-3.065251	-1.769723	-1.002404

N	3.065251	-1.769723	-1.002404
N	2.029661	1.171825	1.416757
N	0.000000	-2.343650	1.416757
N	-2.029661	1.171825	1.416757
N	-2.875232	1.660016	1.912123
N	0.000000	-3.320032	1.912123
N	2.875232	1.660016	1.912123
N	0.000000	0.000000	-3.551861
N	0.000000	0.000000	-2.456111

$Ca(N_2)_7$ (C_1, T)

E=-1444.33278673 au

0 3

Ca	0.007025	0.035143	0.000467
N	-1.469872	0.513243	-1.896683
N	2.232578	0.586451	-0.948476
N	0.246624	2.428044	0.504928
N	-2.129296	0.747996	-2.744332
N	3.216854	0.835861	-1.360274
N	0.353505	3.497252	0.731584
N	1.513361	-0.403208	1.879543
N	0.390858	-1.733733	-1.713470
N	-2.165481	0.633255	1.052039
N	-0.757476	-2.058915	1.117897
N	2.196273	-0.578633	2.723388
N	-3.121701	0.910720	1.508975
N	0.563100	-2.503084	-2.474190
N	-1.089396	-2.975657	1.617739

$[Ca(N_2)_7]^+$ (C_1, D)

E = -1444.1692172 au

Ca	0.045825000	0.002924000	-0.034665000
N	1.806804000	1.639306000	-0.800425000
N	-1.291791000	1.308768000	1.676335000
N	2.542750000	2.366711000	-1.141203000
N	-1.811190000	1.896496000	2.432961000
N	1.895750000	-0.174199000	1.682060000
N	2.664765000	-0.253740000	2.449197000
N	-1.808219000	-2.368854000	1.981111000
N	2.540143000	-2.085906000	-1.601566000
N	-1.289847000	-1.634717000	1.364705000
N	1.804782000	-1.442647000	-1.119514000
N	-1.492411000	-1.308028000	-1.552599000
N	-2.108909000	-1.890740000	-2.237390000
N	-1.485773000	1.611086000	-1.243534000
N	-2.097781000	2.328111000	-1.791096000

$[Sr(N_2)_7]^+$ (C_1, D)

E = -797.2360943 au

Sr	0.086620000	-0.001033000	0.029447000
N	2.035153000	-1.638807000	0.932442000
N	-1.530887000	-1.495465000	-1.548370000
N	2.812195000	-2.308370000	1.297954000

N	-2.134442000	-2.121754000	-2.204227000
N	2.056401000	0.015361000	-1.821584000
N	2.837977000	0.023287000	-2.579629000
N	-2.130633000	2.161023000	-2.167626000
N	2.812483000	2.283650000	1.337929000
N	-1.528327000	1.522585000	-1.522411000
N	2.035550000	1.620841000	0.960084000
N	-1.610014000	1.503019000	1.496703000
N	-2.255685000	2.129184000	2.111659000
N	-1.611718000	-1.526891000	1.471250000
N	-2.258278000	-2.162056000	2.075967000

$[Ba(N_2)_7]^+$ (C_1, D)
E = -792.0194205 au

Ba	0.015476000	0.199276000	-0.002719000
N	2.527831000	0.078234000	-1.532246000
N	-2.478761000	0.417497000	-1.545590000
N	3.470506000	0.026618000	-2.073424000
N	-3.415846000	0.491377000	-2.093910000
N	0.259530000	3.099516000	-0.003384000
N	0.355812000	4.184194000	-0.004051000
N	-3.416618000	0.508355000	2.086420000
N	3.459507000	0.028868000	2.085660000
N	-2.479744000	0.428931000	1.538563000
N	2.519496000	0.080162000	1.539822000
N	-0.198391000	-2.261792000	1.547562000
N	-0.279690000	-3.195183000	2.102104000
N	-0.185948000	-2.271926000	-1.538372000
N	-0.261493000	-3.209057000	-2.087404000

Table S4. Coordinates and energies of the calculated molecules at the M06-2X-D3/cc-pCVTZ-PP level.

$Ca(N_2)_8$ (O_h, T)
E= -913.061602 au
0 3

Ca	0.00000000	0.00000000	0.00000000
N	1.44991300	1.44991300	1.44991300
N	-1.44991300	1.44991300	-1.44991300
N	-1.44991300	-1.44991300	-1.44991300
N	-1.44991300	1.44991300	1.44991300
N	1.44991300	-1.44991300	1.44991300
N	1.44991300	1.44991300	-1.44991300
N	-1.44991300	-1.44991300	1.44991300
N	1.44991300	-1.44991300	-1.44991300
N	2.08235400	-2.08235400	-2.08235400

N 2.08235400 -2.08235400 2.08235400
N -2.08235400 -2.08235400 2.08235400
N -2.08235400 -2.08235400 -2.08235400
N -2.08235400 2.08235400 -2.08235400
N 2.08235400 2.08235400 -2.08235400
N 2.08235400 2.08235400 2.08235400
N -2.08235400 2.08235400 2.08235400

$Sr(N_2)_8$ (O_h, T)
E= -906.944347au
0 3
Sr 0.00000000 0.00000000 0.00000000
N 1.54133400 1.54133400 1.54133400
N -1.54133400 1.54133400 -1.54133400
N -1.54133400 -1.54133400 -1.54133400
N -1.54133400 1.54133400 1.54133400
N 1.54133400 -1.54133400 1.54133400
N 1.54133400 1.54133400 -1.54133400
N -1.54133400 -1.54133400 1.54133400
N 1.54133400 -1.54133400 -1.54133400
N 2.17343400 -2.17343400 -2.17343400
N 2.17343400 -2.17343400 2.17343400
N -2.17343400 -2.17343400 2.17343400
N -2.17343400 -2.17343400 -2.17343400
N -2.17343400 2.17343400 -2.17343400
N 2.17343400 2.17343400 -2.17343400
N 2.17343400 2.17343400 2.17343400
N -2.17343400 2.17343400 2.17343400

$Ba(N_2)_8$ (O_h, T)
E= -901.708315 au
0 3
Ba 0.00000000 0.00000000 0.00000000
N 1.66812000 1.66812000 1.66812000
N -1.66812000 1.66812000 -1.66812000
N -1.66812000 -1.66812000 -1.66812000
N -1.66812000 1.66812000 1.66812000
N 1.66812000 -1.66812000 1.66812000
N 1.66812000 1.66812000 -1.66812000
N -1.66812000 -1.66812000 1.66812000
N 1.66812000 -1.66812000 -1.66812000
N 2.29919100 -2.29919100 -2.29919100
N 2.29919100 -2.29919100 2.29919100
N -2.29919100 -2.29919100 2.29919100
N -2.29919100 -2.29919100 -2.29919100
N -2.29919100 2.29919100 -2.29919100
N 2.29919100 2.29919100 -2.29919100
N 2.29919100 2.29919100 2.29919100
N -2.29919100 2.29919100 2.29919100

$[Ca(N_2)_8]^+$ (D_{4d}, D)
E = -912.896307 au
Ca 0.00000000 0.00000000 0.00000000
N 0.00000000 2.09194400 1.45249300

N -2.09194400 0.00000000 1.45249300
N 0.00000000 -2.09194400 1.45249300
N 1.47922800 -1.47922800 -1.45249300
N 1.47922800 1.47922800 -1.45249300
N -1.47922800 1.47922800 -1.45249300
N 2.09194400 0.00000000 1.45249300
N -1.47922800 -1.47922800 -1.45249300
N 3.00502900 0.00000000 2.04608300
N 2.12487600 -2.12487600 -2.04608300
N 0.00000000 -3.00502900 2.04608300
N -3.00502900 0.00000000 2.04608300
N -2.12487600 2.12487600 -2.04608300
N -2.12487600 -2.12487600 -2.04608300
N 2.12487600 2.12487600 -2.04608300
N 0.00000000 3.00502900 2.04608300

$[Sr(N_2)_8]^+$ (D_{4d}, D)
E = -906.782059 au
Sr 0.00000000 0.00000000 0.00000000
N 0.00000000 2.15999400 1.65733000
N -2.15999400 0.00000000 1.65733000
N 0.00000000 -2.15999400 1.65733000
N 1.52734600 -1.52734600 -1.65733000
N 1.52734600 1.52734600 -1.65733000
N -1.52734600 1.52734600 -1.65733000
N 2.15999400 0.00000000 1.65733000
N -1.52734600 -1.52734600 -1.65733000
N 3.04321300 0.00000000 2.29440100
N 2.15187700 -2.15187700 -2.29440100
N 0.00000000 -3.04321300 2.29440100
N -3.04321300 0.00000000 2.29440100
N -2.15187700 2.15187700 -2.29440100
N -2.15187700 -2.15187700 -2.29440100
N 2.15187700 2.15187700 -2.29440100
N 0.00000000 3.04321300 2.29440100

$[Ba(N_2)_8]^+$ (D_{4h}, D)
E = -901.550325 au
Ba 0.00000000 0.00000000 0.00000000
N 0.00000000 2.26076000 1.89107000
N 0.00000000 2.26076000 -1.89107000
N 2.26076000 0.00000000 -1.89107000
N 2.26076000 0.00000000 1.89107000
N -2.26076000 0.00000000 1.89107000
N 0.00000000 -2.26076000 1.89107000
N 0.00000000 -2.26076000 -1.89107000
N -2.26076000 0.00000000 -1.89107000
N 3.09619000 0.00000000 -2.58824800
N 0.00000000 3.09619000 -2.58824800
N 0.00000000 3.09619000 2.58824800
N -3.09619000 0.00000000 2.58824800
N 0.00000000 -3.09619000 2.58824800
N 3.09619000 0.00000000 2.58824800

N -3.09619000 0.00000000 -2.58824800<br>N 0.00000000 -3.09619000 -2.58824800
Ba$(N_2)_7$ (C_{3v}, T)<br>E= -792.156815 au<br>0 3<br>Ba 0.00000000 0.00000000 0.33121700<br>N 0.00000000 -2.70985400 1.32401600<br>N -2.34680200 1.35492700 1.32401600<br>N 2.34680200 1.35492700 1.32401600<br>N 3.19162900 -1.84268800 -1.10720000<br>N 0.00000000 3.68537600 -1.10720000<br>N -3.19162900 -1.84268800 -1.10720000<br>N 0.00000000 2.66793300 -0.70393700<br>N -2.31049800 -1.33396700 -0.70393700<br>N 2.31049800 -1.33396700 -0.70393700<br>N 0.00000000 0.00000000 -3.60936200<br>N -3.25195000 1.87751400 1.64531200<br>N 0.00000000 -3.75502800 1.64531200<br>N 3.25195000 1.87751400 1.64531200<br>N 0.00000000 0.00000000 -2.51494200
Sr$(N_2)_7$ (C_{3v}, T)<br>E= -797.390477 au<br>0 3<br>Sr 0.00000000 0.00000000 0.18881800<br>N 0.00000000 2.49800500 -0.66314800<br>N 2.16333600 -1.24900200 -0.66314800<br>N -2.16333600 -1.24900200 -0.66314800<br>N 0.00000000 3.54088700 -1.00258600<br>N -3.06649800 -1.77044400 -1.00258600<br>N 3.06649800 -1.77044400 -1.00258600<br>N 2.02934200 1.17164100 1.41669500<br>N 0.00000000 -2.34328200 1.41669500<br>N -2.02934200 1.17164100 1.41669500<br>N -2.87665000 1.66083500 1.91058000<br>N 0.00000000 -3.32166900 1.91058000<br>N 2.87665000 1.66083500 1.91058000<br>N 0.00000000 0.00000000 -3.55270100<br>N 0.00000000 0.00000000 -2.45693600
Ca$(N_2)_7$ (C_1, T)<br>E= -803.511502 au<br>0 3<br>Ca 0.00759300 0.02180400 -0.02565900<br>N -1.37054400 -0.77815000 -1.88230100<br>N 2.27428300 -0.45705100 -0.87820400<br>N 0.53418500 2.07516300 -1.25243300<br>N -1.98249000 -1.12298600 -2.72692500<br>N 3.28268000 -0.64548200 -1.26448700<br>N 0.77425800 2.99422700 -1.80293100<br>N 1.44441900 0.93307600 1.72786100<br>N 0.16254300 -2.45763900 -0.00488700<br>N -2.09512600 1.31124100 0.16802100

N -0.97115900 -0.66902200 2.15378100<br>N 2.09798800 1.35717700 2.50286000<br>N -3.01639800 1.90141800 0.23193400<br>N 0.24019400 -3.55053300 -0.02158100<br>N -1.39652600 -0.95373800 3.12260400
$[Ca(N_2)_7]^+$ (C_1, D)<br>E = -803.346920 au<br>Ca -0.03546400 0.00323400 -0.04890500<br>N -0.80012000 1.65261100 -1.78838300<br>N 1.65955100 1.31223000 1.30535900<br>N -1.13422200 2.36806900 -2.53874300<br>N 2.40613300 1.89497600 1.84368200<br>N 1.70592800 -0.15235300 -1.86709300<br>N 2.46425900 -0.22125700 -2.64559800<br>N 1.98142300 -2.36205300 1.81180800<br>N -1.56402500 -2.07460200 -2.56680100<br>N 1.36574900 -1.63482800 1.28378700<br>N -1.10043700 -1.44580500 -1.80766500<br>N -1.55922800 -1.32939600 1.46096000<br>N -2.23842500 -1.91814700 2.07724100<br>N -1.26764700 1.59747300 1.47483500<br>N -1.81761100 2.30384100 2.09633900
$[Sr(N_2)_7]^+$ (C_1, D)<br>E = -797.231521 au<br>Sr -0.02939000 0.00067900 -0.08722800<br>N -0.93705900 1.63318100 -2.04107000<br>N 1.54622600 1.50599300 1.52898400<br>N -1.30282400 2.29898300 -2.82130500<br>N 2.19962600 2.13443100 2.13297900<br>N 1.82539500 -0.01602200 -2.05671800<br>N 2.58294200 -0.02378400 -2.83886100<br>N 2.17363900 -2.15200700 2.14034100<br>N -1.33135300 -2.29498900 -2.81013900<br>N 1.52775500 -1.51778700 1.53430100<br>N -0.95678100 -1.63009900 -2.03331300<br>N -1.49652400 -1.50493300 1.61416100<br>N -2.10914700 -2.13137300 2.26180200<br>N -1.47848100 1.52950600 1.60847400<br>N -2.08386800 2.16521700 2.25388800
$[Ba(N_2)_7]^+$ (C_1, D)<br>E = -792.001186 au<br>Ba 0.01653800 0.19998600 -0.00259500<br>N 2.52580900 0.08939200 -1.53815800<br>N -2.48137700 0.41116500 -1.54292700<br>N 3.47172000 0.04063200 -2.07373800<br>N -3.42371900 0.48192300 -2.08241700<br>N 0.24544700 3.09770500 -0.00282100<br>N 0.33383600 4.18301600 -0.00341300<br>N -3.42340700 0.49597900 2.07672600<br>N 3.46214900 0.04292600 2.08417200

N -2.48131200 0.42055700 1.53746600
N 2.51884600 0.09214900 1.54403700
N -0.18794400 -2.26459900 1.54611000
N -0.26480600 -3.20207500 2.09425800
N -0.17760700 -2.27386100 -1.53786400
N -0.24993800 -3.21480000 -2.08067000
